# Introducing Concurrent Imaging and Unidimensional Analytics for Plant Stress Responses

**DOI:** 10.3390/plants15030428

**Published:** 2026-01-30

**Authors:** Rubi Quiñones, Francisco Muñoz-Arriola, Sruti Das Choudhury, Ashok Samal

**Affiliations:** 1 Department of Computer Science, Southern Illinois University Edwardsville, Edwardsville, IL 62026, USA; 2 School of Computing, University of Nebraska-Lincoln, Lincoln, NE 68588, USA; s.d.choudhury@unl.edu (S.D.C.); samal@unl.edu (A.S.); 3 School of Natural Resources, University of Nebraska-Lincoln, Lincoln, NE 68588, USA; fmunoz@unl.edu; 4 Department of Biological Systems Engineering, University of Nebraska-Lincoln, Lincoln, NE 68588, USA

**Keywords:** abiotic stress, computer vision, concurrent imaging, growth state of plants, morphological characteristics, plant phenotype analysis

## Abstract

Advancements in phenotyping technologies, including object imaging, high-throughput monitoring, and soft computing, are pivotal for understanding plant responses to environmental stresses. These technologies enable detailed analyses of morphological, physiological, and structural adaptations under abiotic and biotic stresses, such as drought. Current work using multimodal and multi-perspective image processing methods can capture the essential processes that enhance plant resilience and counteract stress by identifying morphological and biochemical indicators. However, the dynamic and complex nature of plant responses poses multiple challenges for generating precise analytics and descriptors of evolving phenotypes. This work introduces analytics for concurrent imaging, adopting the underlying principle of cosegmentation to create taxonomies for new phenotypes. Here, unidimensional refers to the concurrent analysis of multiple images within a single phenotyping dimension: temporal, modal, or perspective, rather than combining information across dimensions. The proposed unidimensional phenotypes integrate concurrent images within individual temporal, modal, or perspective dimensions to capture dynamic morphological and physiological responses that are not observable with conventional single-image or cumulative metrics. Within a high-throughput imagery production system, these phenotypes enable more nuanced quantification of phenotypic changes, leveraging the strengths of simultaneous image analysis to enhance insight into plant adaptations. This workflow aligns with the investigation of plants’ adaptive strategies under abiotic stress and provides quantitative indicators of plant health under adverse environmental conditions.

## 1. Introduction

The quantification of a plant’s biophysical and physiological phenotypic characteristics depends on complex interactions among its genotypes, the environment, and management [1,2]. Phenotypes are critical metrics that reveal the underlying principles of plant responses to a changing climate and the spatial and temporal patterns of variability [3,4]. This information is essential for improving plant resilience and evolution and for securing food production and environmental services for a growing human population [5]. High-throughput plant phenotyping platforms enable rapid, non-invasive imaging and analysis of large plant populations under diverse conditions [6,7]. This capability enables repeated phenotypic measurements throughout the plant life cycle, providing comprehensive longitudinal data on growth and stress responses.

Despite these advances, most phenotyping approaches rely on single-image or single-time-point analyses, which provide only limited insight into dynamic morphological and physiological processes [1,8]. While foundational, these methodologies inherently limit data richness by adopting a static, isolated view of complex biological phenomena. In addition, many phenotypes are tailored to specific crop architectures, reducing their transferability across species with contrasting canopy organization. Plant growth dynamics and multifaceted phenotypic expressions therefore demand analytical approaches that explicitly account for spatial heterogeneity and temporal reorganization.

To address these limitations, this study introduces a concurrent image-based phenotyping framework that leverages relationships within a single phenotyping dimension to capture dynamic plant growth and decay. This methodology aims to develop a taxonomy of dynamic, morphological, and physiological phenotypes, which are then evaluated in two morphologically and physiologically distinct crop species grown under control and drought conditions.

In this study, we define unidimensional phenotyping as the concurrent analysis of multiple images that vary along exactly one phenotyping axis: temporality, modality, or perspective, while all other axes are held fixed. Unlike conventional multivariate or temporal phenotyping frameworks, which integrate information across multiple dimensions simultaneously, the proposed approach isolates within-dimension relationships to generate interpretable descriptors of growth dynamics (temporality), multimodal physiological coupling (modality), and architectural organization (perspective). By emphasizing concurrent image analysis within a single phenotyping dimension, this framework provides a systematic and controlled means of characterizing plant responses without conflating variation across time, modality, and viewpoint.

This work systematically leverages the concurrency within individual dimensions to reveal a dynamic structure that is not accessible through single-image or cumulative descriptors, instead of simply increasing phenotypic dimensionality. The results demonstrate that temporality phenotypes capture dynamic stress signatures, perspective phenotypes reveal species- and treatment-dependent geometric organization, and modality phenotypes expose multimodal relationships that might not be detectable from any single imaging channel. These findings show that even relatively simple unidimensional metrics could expose biologically meaningful patterns in plant development and stress response when derived from concurrent imagery.

The proposed phenotypes are intended to complement, rather than replace, multivariate or cross-dimensional analyses in biological scenarios where interactions among time, modality, and perspective are inherently coupled. Within this scope, this study makes the following contributions:

- We introduce a taxonomy of unidimensional plant phenotypes derived from concurrent image analysis that quantifies basic growth, decay, structural organization, and physiological relationships along temporal, modal, and perspective dimensions.
- We present two curated image datasets: the Single-Dimension Modality Imagery Dataset (SIMID) and the Single-Dimension Perspective Imagery Dataset (SIPID), designed to support the development and evaluation of concurrent image–based phenotypes under control and drought-stress conditions.
- We establish baseline performance by applying existing phenotypes to these datasets, demonstrating the limitations of conventional single-image and cumulative phenotyping approaches.
- We evaluate the efficacy of the proposed phenotypes through comparative analyses across species and stress treatments, highlighting their ability to capture dynamic morphological and physiological behavior enhancing those that could be observable with traditional methods.

## 2. Background

This section reviews computational methods and key taxonomies that enable correlations between phenotypes and genotypes, with a focus on structural, physiological, and morphological traits. State-of-the-art structural and temporal phenotyping primarily uses visible-light imagery [9,10,11] to characterize plant growth and change, offering a sequential, multi-modal view of plant development. These approaches leverage temporal image sequences, analyzing each image to extract normalized feature sets across dimensions, which are then synthesized into a unified analysis. Perspective phenotypes [9,12] have also been developed to describe the overall architectural shape of plants, often contingent on rotational symmetry around a central axis. In addition, physiological data have been extracted from alternative modalities such as fluorescence [13] and infrared imagery [14]. Despite their potential, these modalities have often yielded limited insights into overall plant growth performance, largely due to challenges in calibration, experimental design, and the lack of systematic analytical frameworks that integrate modality-specific signals.

Numerous image processing techniques have been developed to extract color [15], texture [15,16], and shape properties [17] from temporal sequences of visible-light images, facilitating the design of comprehensive phenotypes. Among the earliest notable efforts in this area was the development of a taxonomy by Choudhury et al. [17] to categorize holistic and component phenotypes in the vegetative stage of sorghum plants. This foundational framework captured both detailed component traits and broader holistic descriptors, offering critical insights into plant morphology and development. Building on this work, a second taxonomy [8] was introduced a year later for maize plants, categorizing phenotypes into structural, physiological, and temporal dimensions. This evolution in phenotyping taxonomy underscored the need for advanced dimensional approaches to capture complex plant interactions across time and changing environments.

While multi-modal and temporal approaches have expanded understanding of complex plant traits, it is essential to examine how phenotyping taxonomies and imaging techniques have evolved to capture plant structure, physiology, and temporal changes. This conjecture advances phenotyping taxonomies and their applications in three directions: modal and temporal imaging techniques, AI-driven segmentation and cosegmentation techniques, and existing high-dimensional datasets.

### 2.1. Current Modal and Temporal Approaches

Efforts in plant science have leveraged multidisciplinary approaches to correlate phenotypes with genotypes, employing advanced computational analyses to decipher structural [17], physiological [18,19], and morphological characteristics [18,20], aiming to address critical global food security challenges [2,4,5,8,19,21]. However, reliance on visible light imagery limits exploration of alternative modalities, such as fluorescence [22,23,24], infrared [23], and hyperspectral imaging [23,25], despite their potential to reveal detailed insights into plant stress levels and biochemical makeup [26]. This is due to image-processing issues such as low resolution and color homogeneity between the foreground and the background. Moreover, incorporating perspective in imagery and temporal analyses enhances our understanding of spatial relationships [17,22], object reconstruction [27,28], and change over time [17,22,29], significantly benefiting plant phenomics. These methods also face processing challenges due to high computational costs, the complexity of handling discrepancies across various angles, and the need for consistent data collection over time to accurately model environmental and developmental plant changes. While acknowledging current gaps in the literature for within-dimension analytics (i.e., modality, temporality, and perspective), we advocate a proactive exploration of alternative analytical methods to enrich our interpretive capacity for object changes, using the data already available to us. Building on the advancements in phenotyping taxonomies and imaging techniques, recent advancements in artificial intelligence have further transformed plant phenotyping capabilities. The next section delves into these advancements, particularly focusing on concurrent-imaging techniques and their role in enhancing segmentation accuracy and data synthesis.

### 2.2. Advances in Concurrent-Imaging Techniques for Plant Phenotyping

**Figure 1:**
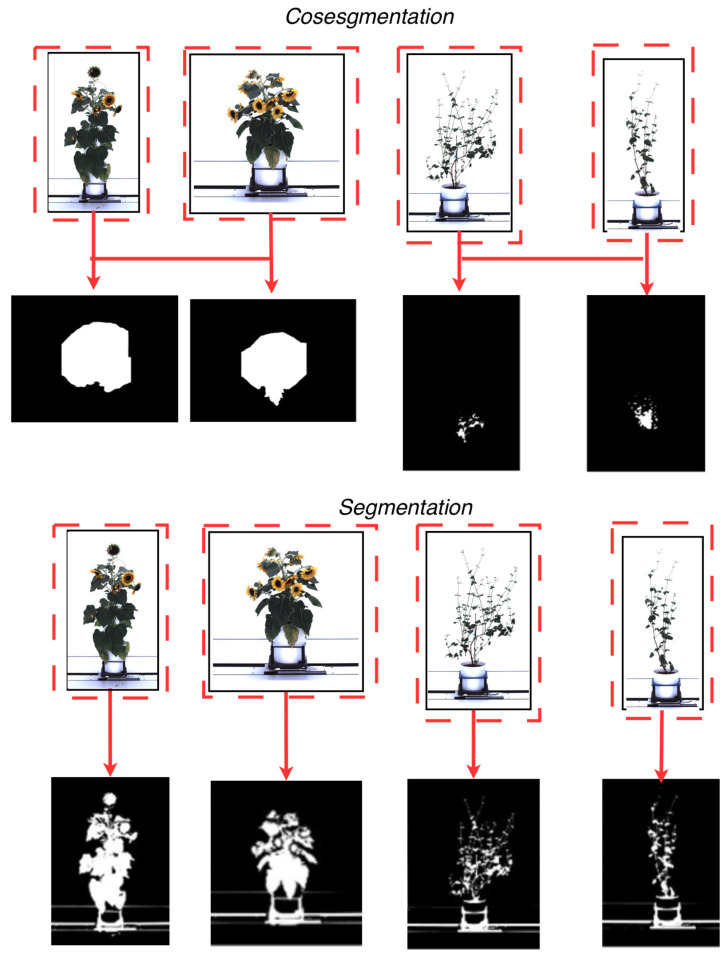
A visual representation of the difference between cosegmentation (**top**), which leverages a collection of images to improve segmentation masks, and segmentation (**bottom**), which derives segmentation masks utilizing one image at a time. The red dashed boxes and arrows indicate how corresponding objects across multiple images are jointly considered and linked to produce shared or mutually informed masks in cosegmentation, whereas in standard segmentation each image is processed independently, yielding masks derived from a single input without cross-image interaction.

Recent advances in artificial intelligence algorithms have improved segmentation in plant phenotyping, enabling precise separation of the plant (foreground) from its background. Current segmentation methods can process high-dimensional data [30] and are semi-supervised [31,32] using superpixel-based methods [33,34], active contour models [35,36], graph-cut formulations [37], and other region-evolution and energy-minimization techniques. These approaches are well established in the computer vision literature and are incorporated into some plant phenotyping workflows. These methods have also shown efficacy in achieving high segmentation accuracy with large datasets needed for proper statistical analysis. The effectiveness of these segmentation methods largely depends on minimizing object overlap to mitigate the risk of plant pixel loss (i.e., missing segmented information). Cosegmentation [38] is a deep learning technique developed to address the potential loss of plant pixel information. This technique distinguishes itself by processing multiple images simultaneously, in contrast to traditional artificial intelligence segmentation techniques that process a single image sequentially (single-image segmentation). Figure 1 shows the difference between a segmentation technique [39] and a cosegmentation framework [40] using plants from the CosegPP dataset [6]. This figure illustrates Otsu’s thresholding [39], a segmentation technique widely used in plant-imaging pipelines, especially when paired with preprocessing steps such as histogram-based enhancement or multilevel thresholding. Recent studies have shown the usefulness of Otsu thresholding within enhanced frameworks (e.g., [39,40,41,42]), so we include it here only as a familiar traditional baseline to visually contrast single-image segmentation with multi-image cosegmentation. Cosegmentation has been shown to improve segmentation accuracy by leveraging dataset information by processing multiple images along a single dimension, such as perspective [43] and species type [40], and extending to multi-dimensional analytics, encompassing species types, perspectives, and other physical characteristics [6].

A cosegmentation application, OSC-CO^2^ [7], was the first to apply advanced deep learning techniques to plant phenotyping analytics using high-dimensional datasets. The results showed that OSC-CO^2^ outperformed other state-of-the-art segmentation and cosegmentation methods, improving overall segmentation accuracy by 3% to 45%. This advancement highlights the ability to improve segmentation precision through cosegmentation in the plant science domain. The premise is to leverage cosegmentation principles to design phenotypes that extract information by processing multiple images along a dimension, providing new insights into plant architectural growth and decay and biochemical changes under different environmental conditions. Moreover, phenotypes developed through this methodology are envisioned to be applicable across a diverse array of species, thereby overcoming the limitations of current phenotyping strategies, which are confined to specific plant architectures. This shift toward a more inclusive and versatile phenotyping approach holds promise for a wide range of applications in plant science research. At the center of this application expansion are data that span multiple imaging dimensions, such as time, modality, and perspective, enabling the design of phenotypes that are both scalable and transferable across species. These data serve not only as input for deep learning models, such as cosegmentation, but also as the foundation for developing high-resolution, interpretable phenotype taxonomies.

### 2.3. Existing Image-Based High-Dimensional Plant Datasets

Table 1 summarizes various existing plant phenotyping datasets, each characterized by different temporalities, image views, modalities, species, and environmental conditions. Notably, datasets such as Panicoid-Phenomap-1 [9], UNL-CPPD [17], and UNL-3DPPD [27] focus on maize and use visible-light imagery captured at specific time intervals, offering insights into the structural and developmental stages of plants under controlled conditions. The FlowerPheno Dataset [44] and CosegPP [6] extend this concept with multi-temporal and multi-modal data, including visible, infrared, and fluorescence modalities. CosegPP, for example, captures phenotypic data for buckwheat and sunflower under both control and drought-stress conditions, providing a broader perspective on stress-induced morphological changes. Other datasets, such as the Leaf Segmentation Challenge [45] and Michigan State University Plant Imagery [46], focus on top-view imaging of specific species, including Arabidopsis, tobacco, and bean, leveraging various modalities such as fluorescence and visible-depth imagery to analyze plant structure and physiology.

While these datasets contribute significantly to advancing plant phenotyping research, critical gaps remain that necessitate the inclusion of new datasets, such as the SIMID and SIPID. Current datasets often rely heavily on single-image modalities or perspectives, limiting the depth of phenotypic characterization and hindering understanding of complex plant behaviors across diverse environmental conditions. For instance, while multi-modal datasets such as CosegPP and Michigan State University Plant Imagery offer valuable insights, they primarily analyze images sequentially without fully exploring interactions, dependencies, or correlations within individual dimensions.

Despite substantial advances in high-throughput plant phenotyping, current computational approaches remain constrained by reliance on single-image or sequential image analysis, limiting their ability to capture dynamic structural reorganization and cross-modal physiological interactions. Many widely used phenotypes summarize plant state with cumulative or static descriptors, such as projected area, aspect ratio, or single-modality intensity, obscuring localized growth, decay, occlusion, and stress-induced decoupling among structural, thermal, and biochemical signals. Furthermore, temporal, modal, and perspective dimensions are typically analyzed independently, without systematically exploiting relationships within a single dimension. These limitations reduce interpretability, restrict transferability across species with contrasting architectures, and hinder robust characterization of plant responses to abiotic stress. Addressing these gaps requires phenotypes explicitly designed to leverage concurrent images within a single dimension, enabling direct quantification of spatial, geometric, and physiological change that cannot be captured through isolated or cumulative measurements.

## 3. Materials and Methods

### 3.1. Dataset for Single-Dimension Phenotypes

The SIMID dataset is designed to evaluate the efficacy of unidimensional modality phenotypes. The dataset (Table 2) includes images from three modalities: visible, fluorescence, and infrared, which are the most commonly used modalities for plant phenotyping. It contains data for 12 plants: 6 *Fagopyrum esculentum* (buckwheat) samples (3 control and 3 drought-induced) and 6 *Helianthus annuus* L. (sunflower) samples (3 control and 3 drought-induced). Each buckwheat sample includes seven images spanning the plant’s growth from seedling to maturity, while each sunflower sample includes fourteen images covering the same growth stages. Each plant was imaged from a fixed side angle (0°) in three modalities (visible, infrared, fluorescence). The buckwheat samples have fewer images because the buckwheat life cycle is significantly shorter than that of the sunflower. Although only three biological replicates per species × treatment are available in SIMID, repeated measurements over time and across modalities provide sufficient effective sample size to estimate phenotype dynamics; however, limited biological replication increases variance and reduces power for population-level inference, particularly for nonlinear and event-based phenotypes.

The SIPID dataset is used to investigate the efficacy of unidimensional perspective phenotypes. The dataset (Table 2) contains binary images from the visible modality taken from four side-view angles: 0°, 72°, 144°, and 216°. It includes data for 28 plants: 14 buckwheat samples: 8 control and 6 drought-induced, and 14 sunflower samples: 7 control and 7 drought-induced. The plants are imaged from the seedling stage through to maturity.

**Figure 2:**
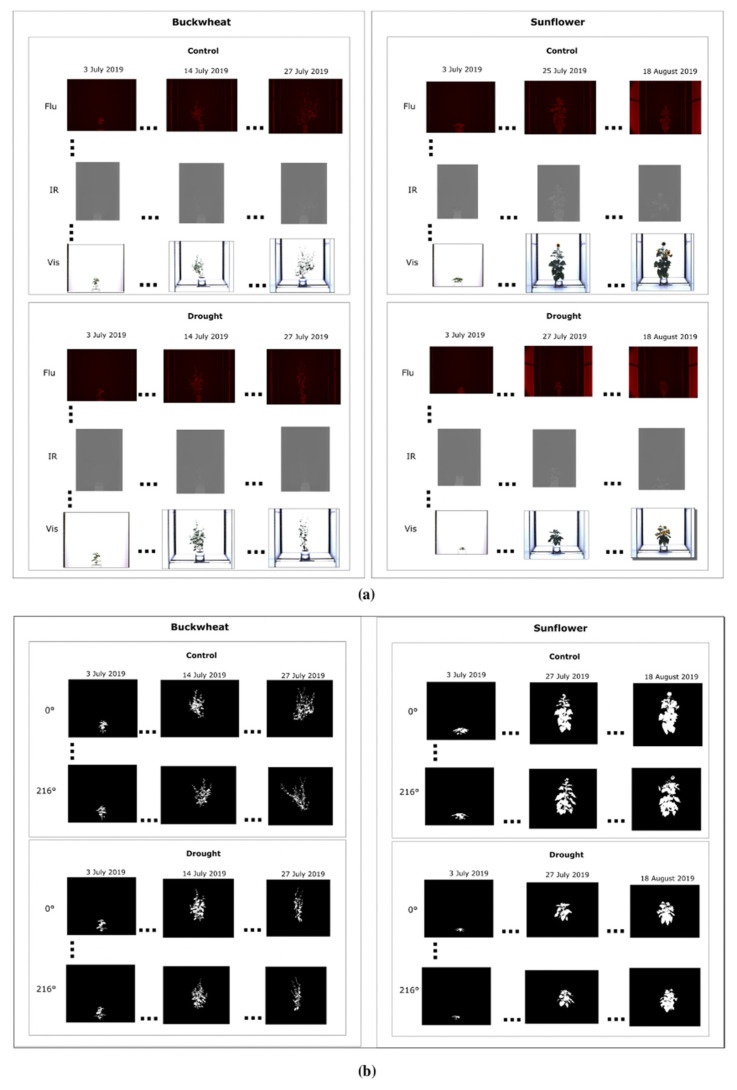
Sample images of buckwheat and sunflower from the SIMID (**a**) and SIPID (**b**) dataset repositories, showcasing three different modalities and various side-view perspectives, respectively.

The figure below presents a subset of images illustrating the visual appearance of the SIMID dataset (Figure 2a) and the SIPID dataset (Figure 2b). These datasets are freely downloadable from 10.5281/zenodo.17400167.

### 3.2. Plant Materials and Imaging Acquisition Setup

Buckwheat (*Fagopyrum esculentum*) and sunflower (*Helianthus annuus* L.) were selected as focal species for their distinct morphological architectures. Buckwheat, with its fine foliar structure and branching, contrasts with sunflower’s upright stature and large, broad leaves and inflorescences. This contrast provides a diverse range of phenotypes for validating the proposed unidimensional phenotypes. To evaluate the generalizability of these phenotypes, we conducted a comparative study using both species under controlled and stress conditions. The goal was to demonstrate the precision, scalability, and applicability of the phenotypes across divergent plant architectures and environmental responses.

Image data were acquired using the LemnaTec Scanalyzer system at the University of Nebraska–Lincoln (Lincoln, NE, USA) a high-throughput phenotyping platform equipped with automated multi-modal and multi-angle image capture capabilities with controlled illumination, fixed camera geometry, and a uniform white. background. This setup eliminates cast shadows and minimizes variability due to lighting conditions across time points, modalities, and viewpoints. As a result, no additional illumination normalization or shadow correction was required. Plants were grown under controlled environmental conditions, with both species divided into two treatment groups: well-watered controls and drought-induced stress. For the SIMID dataset, six plants of each species were used: three per treatment group, resulting in a total of 12 plants. Each plant was imaged across three modalities: visible light, infrared, and fluorescence. Imaging was limited to a single fixed side-view angle (0°) but repeated across 7 to 14 time points to capture phenotypic changes over time yielding 21 images per buckwheat plant and 42 images per sunflower plant. This configuration enables the analysis of modality-specific stress indicators while holding perspective constant.

In contrast, the SIPID dataset was curated to investigate structural and architectural variation through multi-angle imaging. It included 14 buckwheat plants (8 control, 6 drought) and 14 sunflower plants (7 control, 7 drought). All SIPID images were captured in the visible modality only but from four distinct side-view angles: 0°, 72°, 144°, and 216°. Like SIMID, each plant was imaged over 7 to 14 time points yielding 28 image per buckwheat plant and 56 images per sunflower. This setup facilitates the development of phenotypes sensitive to perspective-driven differences, such as symmetry, occlusion, and shape deformation, while keeping modality and time point fixed.

Both SIMID and SIPID were derived from the larger CosegPP image corpus [6] but were curated specifically to support the formulation and validation of concurrent image-based phenotypes. SIMID focuses on phenotype extraction across spectral modalities, while SIPID enables structural phenotype assessment across viewpoints. Together, these datasets provide a structured, multidimensional foundation for developing phenotypes that are both interpretable and generalizable across species, treatments, and imaging configurations.

All proposed phenotypes were derived from ground-truth binary plant masks for SIPID. These masks were manually generated using Adobe Photoshop 2025 by carefully segmenting the plant foreground from the background for each image. This manual segmentation protocol follows established practices used in prior studies (e.g., [6,7]) and ensures that the extracted phenotypes are not confounded by automated segmentation errors. Overlapping leaves and self-occlusion were not treated as noise in this study. Instead, they were considered meaningful biological structures that reflect plant architecture and growth dynamics under stress. Consequently, no morphological filtering or post-processing was applied to artificially suppress overlap or occlusion effects.

### 3.3. Proposed Taxonomy

**Figure 3:**
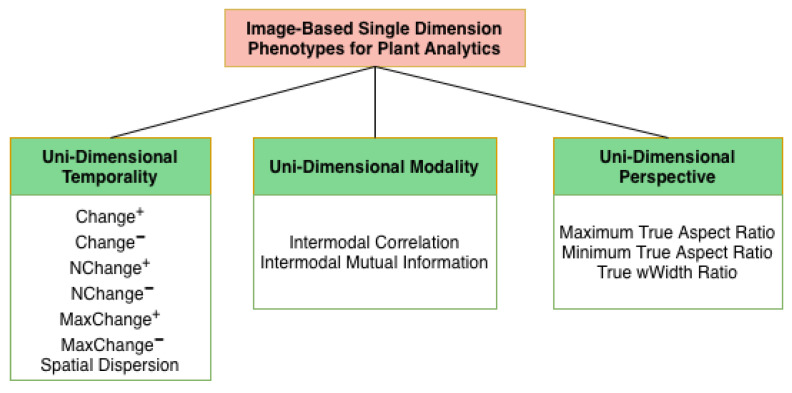
Introduced taxonomy of image-based single dimension phenotypes for plant analytics with a Unidimensional Temporality category, a Unidimensional Modality category, and a Unidimensional Perspective category.

Figure 3 shows the proposed categorical taxonomy of phenotypes for image-based plant analytics. In this work, unidimensional phenotypes are defined as phenotypes derived from the concurrent analysis of multiple images within a single phenotyping dimension: temporality, modality, or perspective, while holding the remaining dimensions fixed. This formulation explicitly differs from multivariate or temporal fusion frameworks, which combine variation across multiple phenotyping axes simultaneously. Here, concurrency is restricted to a single axis, enabling isolation of within-dimension relationships without cross-dimension confounding. Although such analyses operate on multiple images, they are considered unidimensional because they intentionally exploit variation along only one axis at a time, rather than integrating across multiple phenotyping dimensions simultaneously. The phenotypes are classified into three categories: unidimensional temporality, modality, and perspective. Unidimensional Temporality (UDT) phenotypes evaluate the extent of positive and negative growth change, Unidimensional Modality (UDM) phenotypes provide relational information between various modalities, and Unidimensional Perspective (UDP) phenotypes capture the refined view of a plant’s structural architecture. This unidimensional formulation distinguishes the proposed phenotypes from conventional multi-dimensional approaches by isolating and intensively characterizing variation along a single phenotyping axis.

The single-dimension phenotypes can be utilized for their ability to (1) differentiate between a control and an abiotic environment and (2) identify unique patterns of plant growth and decay, aiding in decision-making for enhancing plant resilience. These phenotypes will capture an accurate structural representation of the plant through unidimensional perspective phenotypes, assess plant health by determining relational correlations across modalities using unidimensional modality phenotypes, and identify both the areas and magnitude of plant growth and decay over time using unidimensional temporality phenotypes.

#### 3.3.1. Unidimensional Temporality Phenotypes

We propose 6 UDT phenotypes that quantify plant growth and decay by capturing the spatial distribution of change. These phenotypes account for new phenotypic information that may appear in subsequent images but was obscured in earlier ones due to factors such as leaf occlusion or overlap. These phenotypes can detect both the occurrence and magnitude of growth and decay, thereby providing valuable insights into the plant’s developmental progress and growth rate. They correspond to (i) net structural change magnitude, (ii) spatial fragmentation of change, and (iii) spatial organization of change. Importantly, while these phenotypes are derived from structural change masks, their temporal and spatial patterns reflect physiological regulation of growth processes, including turgor-driven expansion, leaf reorientation due to water potential gradients, and stress-induced suppression of coordinated canopy deployment. Rather than directly measuring physiological variables, Change^+^, Change^−^, NChange^+^, NChange^−^, and Dispersion^+^ act as image-based proxies for how physiological stress manifests through spatially localized or coordinated structural reorganization over time.

**Figure 4:**
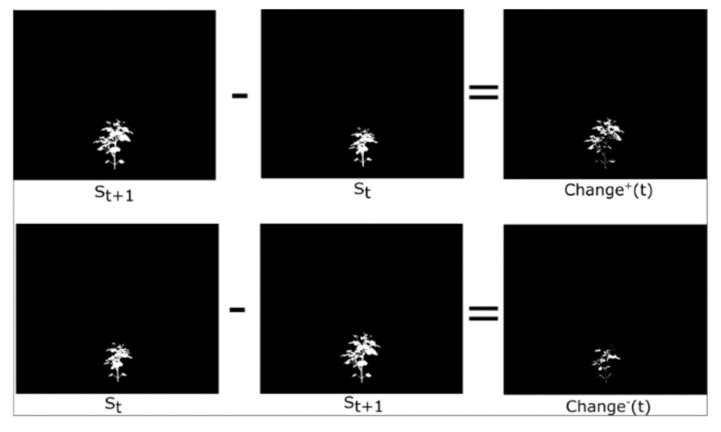
Visual representation of our $Change^{+}$ and $Change^{-}$ phenotype.

Phenotypic changes can be either positive or negative. Change^+^ quantifies additions to the plant between consecutive time points, from time t to time t + 1, representing new growth or visible changes. Conversely, Change^−^ captures the parts of the plant that were present at time t but absent at time t + 1, t, indicating decay or occlusion. The phenotypes are visually illustrated in Figure 4. The estimated Change^+^ and Change^−^ are defined using a sequence of the plant images, $P_{1},\;{\;P}_{2},\;\ldots ,\;P_{n}$ corresponding segmented masks $S_{1},\;{\;S}_{2},\;\ldots ,\;S_{n}$, where $S_{i}\in \{0,1\}$ represents the segmented masks for $P_{i}\;(1\;\leq \;i\;\leq \;n$). To compute the phenotype, we focus on a specific time point t. Below are the equations:

(1) $$Change^{+}\;(t)=max(S_{t+1}-S_{t},0),$$

(2) $$Change^{-}\;(t)={max(S}_{t}-S_{t+1},0),$$

where the max operator is applied element-wise. These definitions ensure that Change^+^ and Change^−^ are binary-valued masks representing newly appearing and disappearing plant regions, respectively, and are therefore suitable for connected component analysis.

After deriving the Change^+^ and Change^-^ phenotypes, we quantify the connected components which are distinct objects in an image. A connected component comprises a set of adjacent pixels connected through 8-connectivity, meaning pixels are considered connected if their edges or corners touch. This includes connections in horizontal, vertical, or diagonal direction. The number of connected components in a Change^+^ image at time point t represents the number of new growth components. Similarly, the number of connected components in a Change^−^ image at time point t indicates the number of decayed or removed components. These quantities are referred to as the NChange^+^ and NChange^+^ and are defined as the number of connected components in the binary Change^+^ and Change^-^ masks, respectively. These quantities represent counts of spatially distinct growth or disappearance events and are not normalized by plant area or mask size. Connected components were identified using 8-connectivity.

(3) $$NChange^{+}\left(t\right)=\left|Connected\left(Change^{+}\left(t\right)\right)\right|,$$

(4) $$NChange^{-}\left(t\right)=\left|Connected\left(Change^{-}\left(t\right)\right)\right|,$$

The MaxChange^+^ and MaxChange^−^ phenotypes are derived from the NChange^+^ and NChange^+^ phenotypes. After quantifying the number of growth and decayed components in each image i at time point t, we then determine the area of the largest change. This involves calculating the area of the largest connected component among the growth components (Change^+^) and separately among the decay components (Change^−^). Below are the equations for calculating the MaxChange^+^ and MaxChange^−^ phenotypes which are defined as the pixel area of the largest connected component in the binary Change^+^ and Change^−^ masks, respectively, and therefore quantify the dominant contiguous growth or disappearance event at each time point.

(5) $$MaxChange^{+}\left(t\right)=\underset{CC_{i}\in \mathrm{CC}s^{+}(t)}{\max}CC_{i}^{t}\;,$$

(6) $$MaxChange^{-}\left(t\right)=\underset{CC_{i}\in \mathrm{CC}s^{-}(t)}{\max}CC_{i}^{t}\;,$$

Let $CCs^{+}(t)=Connected\left(Change^{+}\left(t\right)\right)$ denote the set of connected components representing positive changes at time point t, and $CCs^{-}\left(t\right)=Connected\left(Change^{-}\left(t\right)\right)$ represents those corresponding to negative changes. Each ${CC}_{i}^{t}$ indicates the area of the $i^{th}$ connected component within the respective change category at the time point t.

The Spatial Dispersion phenotype builds on the Change^+^ and Change^−^ phenotype by characterizing the spatial arrangement of detected positive changes at each time point. While the NChange^+^ and NChange^−^ phenotype quantifies the number of connected components, the Dispersion^+^ phenotype captures their spatial distribution. Specifically, Dispersion^+^ is computed by extracting the centroids of all connected components in the Change^+^ mask and summarizing the spatial distribution of these centroids using pairwise Euclidean distances. The average of these distances across all components provides a scalar descriptor of spatial scattering at time

(7) $$Dispersion^{+}\left(t\right)=\frac{\sum_{CC_{i}\in CCs^{+}(t)}distance(CC_{i},\;nearest\left(CC_{i}\right))}{\left|Connected\left(Change^{+}\left(t\right)\right)\right|}\;,$$

where $CCs^{+}(t)=\;Connected\left(Change^{+}\left(t\right)\right)$ represents the set of connected components extracted from the binary mask of positive change at time t, and distance [$CC_{i},\;nearest\left(CC_{i}\right)]$ is the Euclidean distance between the centroid of the component $CC_{i}$ and the centroid of its nearest neighboring component. A low Dispersion^+^ value implies that changes are spatially clustered, while higher values indicate a more dispersed distribution of change across the plant structure. Biologically, clustered change is consistent with localized growth or reorientation events (e.g., within leaf clusters or branching points), whereas high Dispersion^+^ reflects canopy-wide coordination or simultaneous response across multiple organs. Under drought stress, increased Dispersion^+^ can indicate loss of coordinated growth regulation, consistent with heterogeneous turgor loss and spatially variable hydraulic constraints within the canopy.

The development of the spatial Dispersion^+^ phenotype required several machine learning–based image-processing techniques. First, connected component labeling is applied to segment individual regions of change, a fundamental operation in morphological image analysis often supported using region-growing or graph-based segmentation algorithms. Centroids are computed using region properties extraction, and the nearest neighbor is identified via a k-nearest neighbors (k-NN) search algorithm, a commonly used method in spatial reasoning and unsupervised learning. These methods collectively allow the phenotype to transform high-dimensional, time-indexed image data into an interpretable scalar descriptor.

As a proof-of-concept, Spatial Dispersion^+^ demonstrates how machine learning and computer vision techniques can be embedded directly into phenotype design, rather than used solely for post hoc analysis. Its inclusion in the taxonomy of unidimensional temporality phenotypes opens new opportunities for interpreting plant stress responses in terms of spatial dynamics, offering a new layer of insight into plant growth adaptation mechanisms under varying environmental conditions. Hereafter, we refer to these UDT phenotypes collectively as Change^+^, Change^−^, NChange^+^, NChange^−^, MaxChange^+^, MaxChange^−^, and Dispersion^+^, when not discussing the phenotypes by their time-index.

#### 3.3.2. Unidimensional Perspective Phenotypes

We propose three UDP phenotypes that leverage perspective imagery to capture a more accurate representation of the plant’s shape architecture. In high-throughput phenotyping systems, it is often possible to image the plant from various angles. We hypothesize that, in cases where the plant is not rotationally symmetric, the degree of asymmetry can be effectively captured by examining its multiple viewpoints.

Given a set of n perspective images of a plant at a specific time point $P=\{P_{1},P_{2},\ldots ,P_{n}\}$, where $P_{i}$ is the $i^{th}$ image, we first compute the height and width of the plant. Let $h_{i}$ and $w_{i}$ be the height and width of the plant in the $i^{th}$ view, respectively. We assume that $h_{1}=h_{2}=\cdots =h_{h}=h$, meaning, the height of the plant remains consistent across all images. We then define a set of accurate aspect ratios. Maximum True Aspect Ratio (TARmax) is defined as the ratio of the maximum width of the plant observed in any view to the height of the plant. It is calculated as follows:

(8) $$TAR_{max}=\frac{W_{max}}{h},$$

where $W_{max}=\;\underset{i\leq n}{\max\{}w_{i}\}$. Minimum True Aspect Ratio (TAR_min_) is defined as the ratio of the minimum width of the plant observed in any view to the height of the plant. It is calculated as follows:

(9) $$TAR_{min}=\frac{W_{min}}{h},$$

where $w_{min}=\underset{i\leq n}{\min}{\{w}_{i}\}$. True Width Ratio (TWR) is defined as the ratio of the minimum width of the plant in any view to the maximum width in any view. It is calculated as follows:

(10) $$TWR=\frac{W_{min}}{W_{max}},$$

where $w_{min}=\underset{i\leq n}{\min}{\{w}_{i}\}$, and $w_{max}=\underset{i\leq n}{\max}{\{w}_{i}\}$. These phenotypes employ a broad range of perspectives to more accurately estimate the biological attributes that underlie a plant’s three-dimensional architecture.

#### 3.3.3. Unidimensional Modality Phenotypes

We propose two UDM phenotypes that utilize various modalities (visible, infrared, and fluorescence) to capture the dependency of modal information that is essential in investigating the overall health of a plant. Advanced imaging technologies have been developed to assess both physiological and physical aspects of plants, demonstrating that while a plant may appear visibly healthy, it could be biologically stressed. We introduce two phenotypes, called Intermodal Correlation and Intermodal Mutual Information. These phenotypes have the potential to indicate the degree of relationship between modalities that provide physical, biochemical, and stress-related information.

Given images from any two different modalities $I_{m1}$ and $I_{m2}$, let $B_{m1}$ and $B_{m2}$ be the bounding box images of the plant in the two modalities. Since the images capture the same view of the plant but in different modalities, the plant is aligned in both images, ensuring that the bounding boxes are also aligned. However, the resolutions of these images are likely to differ. Thus, we rescale the image with the higher resolution to match the resolution of the other image. Assuming, without loss of generality, that the size of $B_{m1}$ is greater than $B_{m2}$, we rescale all modality images to a shared reference resolution prior to computing intermodal measures. Specifically, given bounding-box images $B_{m1}$ and $B_{m2}$, both images are resampled using bilinear interpolation to match the spatial resolution of the lowest-resolution modality, yielding $B_{m1}^{\prime }$ and $B_{m2}^{\prime }$. Intermodal Correlation (IC) was computed using Pearson’s correlation coefficient and thus ranges from −1 to 1. Intermodal Mutual Information (IMI) was computed using joint intensity histograms with 64 bins per modality channel. IC and IMI as follows:

(11) $$IC=Correlation(B_{m1}^{\prime },\;B_{m2}),$$

(12) $$IMI=MutualInformation(B_{m1}^{\prime },\;B_{m2})$$

IC measures how well the images of the plant, $B_{m1}^{\prime },\;B_{m2}^{\prime }$, in different modalities are related. A positive correlation (0≤IC≤1) indicates that the modalities influence each other, with a higher correlation signifying a greater degree of interconnection and dependency between the modalities. A negative correlation suggests an inverse relationship, while a correlation of zero indicates no relationship between the modalities. To compute IC, consider the set of overlapping plant pixels between the two images. Let $X={\{x}_{1},\;x_{2},\ldots ,\;x_{n}\}$ be the set of intensity values at the overlapping points in $B_{m1}^{\prime }$, and $Y={\{y}_{1},y_{2},\ldots ,y_{n}\}$ be the corresponding intensity values in $B_{m2}^{\prime }$. Here, n is the number of overlapping points, given by $n=|B_{12}|$. IC is then computed as:

(13) $$IC=Correlation\left(B_{m1}^{\prime },\;B_{m2}\right)=\frac{S_{xy}}{S_{x}S_{y}}$$

where $S_{x}$ is the sample variance of X (the plant image points in the first modality), Sy is the sample variance of Y (the plant points in the second modality), and $S_{xy}$ is the sample covariance.

IMI is based on information theory and measures the degree of mutual dependence between two random variables [48], in this case, the two modalities. It quantifies the amount of information one variable provides about the other. A value of zero in IMI indicates independence between the modalities, while a higher IMI value suggests a significant reduction in uncertainty, implying a stronger shared relationship between the modalities.

To compute IMI, start by determining the set of overlapping plant pixels in both images, $B_{12}=B_{m1}^{\prime }\cap B_{m2}^{\prime }$. Let $X={\{x}_{1},x_{2},\ldots ,x_{n}\}$ be the set of intensity values at the overlapping points in $B_{m1}^{\prime }$, and $Y=\{y_{1},y_{2},\ldots ,y_{n}\}$ be the corresponding intensity values in $B_{m2}^{\prime }$. The number of overlapping points, n, is given by $n=|B_{12}|$. IMI can be computed as:

(14) $$IMI=MutualInformation\left(B_{m1}^{\prime },B_{m2}\right)=H\left(X\right)+H\left(Y\right)-H(X,Y)$$

where H(X) is the entropy of X, H(Y) is the individual entropy of Y, and H(X,Y) is their joint entropy. They are defined below:

(15) $$H\left(X\right)=-\sum_{j=1}^{m}P\left(X=u_{j}\right)\log_{2}P\left(X=u_{j}\right)$$

(16) $$H\left(Y\right)=-\sum_{k=1}^{n}P\left(Y=v_{k}\right)\log_{2}P(Y=v_{k})$$

(17) $$H\left(X,Y\right)=\sum_{j=1}^{m}\sum_{k=1}^{n}P\left(X=u_{j},Y=v_{k}\right)\log_{2}P(X=u_{j,\;}Y=v_{k})$$

Here, m is the number of distinct values in X, and n is the number of distinct values in Y. In our context, X and Y correspond to the images in the two modalities. Given that the spectral resolution in the two modalities may differ (e.g., m≠n), the above descriptions apply to single-band or combined band modality images. For example, in the visible modality, you might use the red, green, and blue bands separately or compute a combined grayscale image. Similarly, you can generate composite images or indices that integrate different bands or modalities to compute phenotypes, helping to understand relationships between various indices, such as the NDVI index [24] and fluorescence bands. This approach allows for the generation of a suite of phenotypes by considering different combinations of modalities or indices.

Together, IC and IMI act as physiologically informed relational phenotypes in our taxonomy. While they do not directly measure physiological quantities such as stomatal conductance or chlorophyll fluorescence efficiency, they quantify the degree of coupling between structural (visible), thermal (infrared), and biochemical (fluorescence) signals. Reductions in intermodal dependency are consistent with stress-induced decoupling between canopy architecture, transpiration-driven cooling, and photosynthetic activity, a phenomenon widely reported during progressive drought stress. IC reflects synchronized changes across modalities (e.g., structural–thermal coupling during drought), while IMI captures shared biochemical–structural information that is inaccessible to any single modality. By combining visible, infrared, and fluorescence measurements, these two phenotypes provide an aggregate representation of plant vigor. High intermodal dependency typically corresponds to consistent physiological functioning, whereas reductions in correlation or mutual information may indicate stress-induced decoupling of structural, thermal, and biochemical processes. These multimodal relationships are now explicitly included in our updated taxonomy figure to highlight their role as physiological traits.

## 4. Results

This section presents a comprehensive evaluation of existing and proposed phenotypes using the SIMID and SIPID datasets. We first assess the performance of conventional phenotypes to establish baseline behavior across temporal, perspective, and modality dimensions. We then report the efficacy of the proposed unidimensional temporality (UDT), perspective (UDP), and modality (UDM) phenotypes, highlighting their ability to capture species-specific architectural strategies and stress-induced morphological and physiological changes within the evaluated broadleaf species. Results are organized by phenotype category to facilitate direct comparison across species and treatment conditions.

### 4.1. Baseline Evaluation Using Conventional Phenotypes

This subsection establishes baseline performance by analyzing widely used conventional phenotypes across temporal, perspective, and modality dimensions that were derived from the LemnaTec Scanalyzer system, a high-throughput phenotyping platform. These results provide a reference point for interpreting the added value of the proposed unidimensional phenotypes and demonstrate the limitations of traditional single-image and cumulative metrics in capturing species-specific stress responses.

#### 4.1.1. Temporality Growth Responses

**Figure 5:**
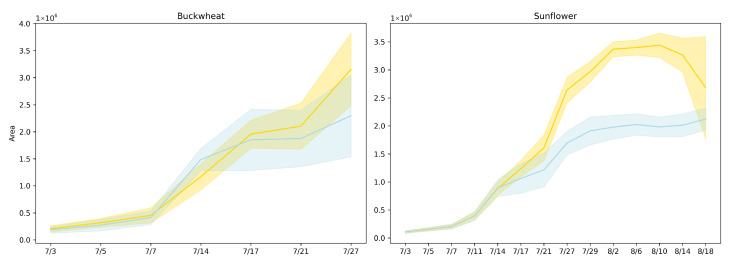
Projected canopy area over time for buckwheat (**left**) and sunflower (**right**) under control (yellow) and drought (blue) conditions. Lines represent mean area across replicates, and shaded regions denote the 95% confidence interval.

Figure 5 presents the plant area [17] (in pixels) over time for both buckwheat and sunflower under control and drought conditions using lines represent mean area across replicates, and shaded regions denote the 95% confidence interval. Drought was induced on 14 July.

Projected canopy area captures net expansion but also reflects how growth is expressed through canopy deployment over time. In buckwheat, the similar magnitudes and essentially parallel trajectories under control and drought conditions indicate that water limitation does not immediately constrain overall canopy development. The modest separation observed late in the experiment suggests delayed or weak stress expression at the population level, implying that buckwheat can preserve projected area through architectural compensation rather than sustained biomass accumulation. The increasing variability among plants toward the end of the season further indicates that drought responses are highly individual-specific, consistent with a species whose growth strategy relies on flexible, fine-scale reorganization rather than coordinated suppression of expansion.

Sunflower exhibits a markedly different response, with drought producing an early, persistent, and magnitude-dependent reduction in projected area. The substantially lower canopy area under drought reflects strong constraints on leaf expansion and biomass accumulation, indicating that sunflower growth is tightly coupled to water availability. The sustained separation between treatments demonstrates that drought imposes a long-lasting limitation on canopy development rather than a transient delay, making projected area a robust discriminator of stress sensitivity for this species. Together, these results highlight that while projected area effectively captures drought-induced growth suppression in sunflower, it provides limited insight into species like buckwheat, where stress responses manifest primarily through structural reorganization rather than changes in net canopy size. This contrast motivates the need for complementary temporal phenotypes that explicitly quantify change dynamics beyond cumulative area.

#### 4.1.2. Perspective Growth Responses

**Figure 6:**
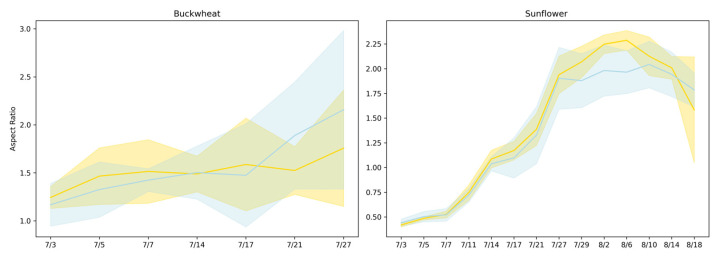
Aspect ratio trajectories for buckwheat (**left**) and sunflower (**right**) under control (yellow) and drought (blue) conditions. Solid lines show the mean aspect ratio across replicates, and shaded regions denote the 95% confidence interval.

Figure 6 presents the aspect ratio [17] trends of buckwheat and sunflower under both control and drought conditions over a period from 3 July to 18 August. Drought was induced on 14 July.

Aspect ratio reflects how plant growth is allocated between vertical elongation and lateral expansion, providing a coarse descriptor of architectural balance under stress. In buckwheat, aspect ratio values remain within a relatively narrow range across time and show substantial overlap between control and drought treatments. The absence of a consistent shift indicates that water limitation does not induce a uniform change in global canopy proportions. Instead, variability in aspect ratio reflects localized stem and leaf reorientation events that alter individual views without producing sustained directional elongation or compaction. This behavior reinforces the interpretation that buckwheat’s response to stress is dominated by fine-scale architectural flexibility rather than coordinated changes in overall geometry.

Sunflower exhibits a clearer, more interpretable architectural response. Aspect ratio increases rapidly during early development, reflecting strong vertical dominance during canopy expansion, and reaches systematically lower peak values under drought. This attenuation indicates constrained vertical growth and altered leaf posture under limited water availability. The relatively tight distributions across replicates suggest that sunflower responds to drought in a coordinated manner, producing a consistent geometric signature of stress. Together, these results show that aspect ratio is informative for species with structured, upright architectures such as sunflower but less effective for species like buckwheat, where flexible morphology and viewpoint sensitivity obscure treatment-level geometric signals.

#### 4.1.3. Modality Growth Responses

**Figure 7:**
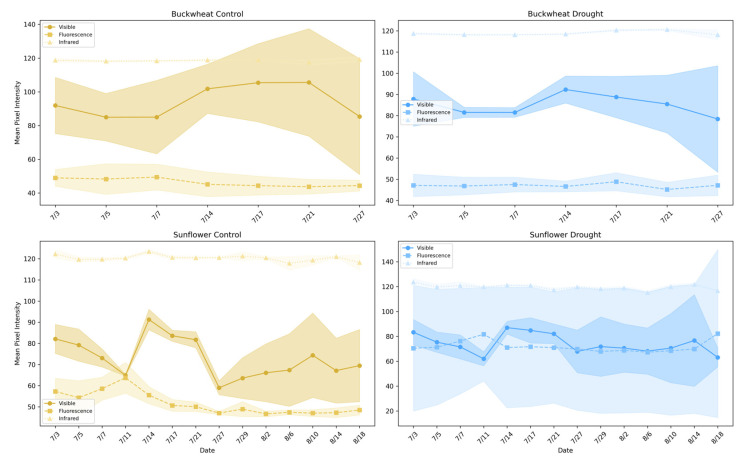
Average pixel intensity trends across visible, infrared, and fluorescence imaging modalities for buckwheat (**top row**) and sunflower (**bottom row**) under control (**left column**) and drought conditions (**right column**).

Figure 7 illustrates the average pixel intensity trends across different imaging modalities (visible, infrared, and fluorescence) for buckwheat (top row) and sunflower (bottom row) under control and drought conditions. This analysis highlights the behavior and limitations of single-modality assessments in capturing plant health and responses to stress.

Single-modality intensity trends provide coarse indicators of plant condition but reflect isolated structural or physiological signals rather than integrated responses. In buckwheat, visible, infrared, and fluorescence intensities exhibit modest temporal variation with substantial overlap between control and drought treatments. The absence of strong separation indicates that drought does not induce a consistent, population-level shift in any single modality. Elevated infrared intensity under drought suggests reduced canopy density and increased background exposure, but this signal remains weak and variable. Fluorescence intensities show limited dynamic range across treatments, implying that stress-related biochemical signals are either subtle or masked by structural heterogeneity. Overall, buckwheat’s single-modality responses reflect localized, plant-specific variability rather than coordinated physiological change.

Sunflower exhibits clearer modality-dependent stress signatures. Under drought, visible-light intensity remains consistently lower, indicating suppressed structural development, while infrared intensity is elevated, reflecting reduced shading and increased thermal exposure. These opposing trends demonstrate that drought alters both canopy structure and energy balance in a coordinated manner. Fluorescence intensity, while comparatively stable, shows reduced variability under drought, consistent with constrained physiological activity during stress. Together, these results show that single-modality phenotypes can capture gross treatment effects in species with coordinated growth patterns, such as sunflower. Still, they fail to reveal how structural, thermal, and biochemical signals interact. This limitation motivates the use of relational, multimodal phenotypes that explicitly quantify cross-modal dependencies, which are explored in the subsequent section.

### 4.2. Evaluation of Proposed Unidimensional Temporal Phenotypes

This study evaluates the efficacy of our single-dimensional phenotypes for temporality, perspective, and modality, focusing on their ability to capture complex plant responses across varying environmental conditions. By analyzing each phenotype dimension separately, we aim to assess their strengths and limitations in providing meaningful insights into plant growth, structural changes, and physiological interactions. This evaluation will determine the types of conclusions that can be drawn from each approach, highlighting how these phenotypes contribute to a comprehensive understanding of plant behavior and adaptation, and identifying areas where integrating across dimensions may further enhance phenotypic characterization.

We focus our study on buckwheat and sunflower, analyzing six UDT phenotype measurements extracted from the dataset images: $Change^{+}$, $Change^{-}$, $NChange^{+}$, $NChange^{-}$, $MaxChange^{+}$, $MaxChange^{-}$, and $Dispersion^{+}$. We used our concurrent imaging approach to assess the phenotypes’ ability to quantify growth and decay in plants by capturing the spatial distribution of change. All statistical analyses treat individual plants as the unit of biological replication, with repeated time-point measurements aggregated at the plant level before between-treatment or between-species comparisons.

#### 4.2.1. Magnitude of Change ($Change^{+}$, $Change^{-}$)

**Figure 8:**
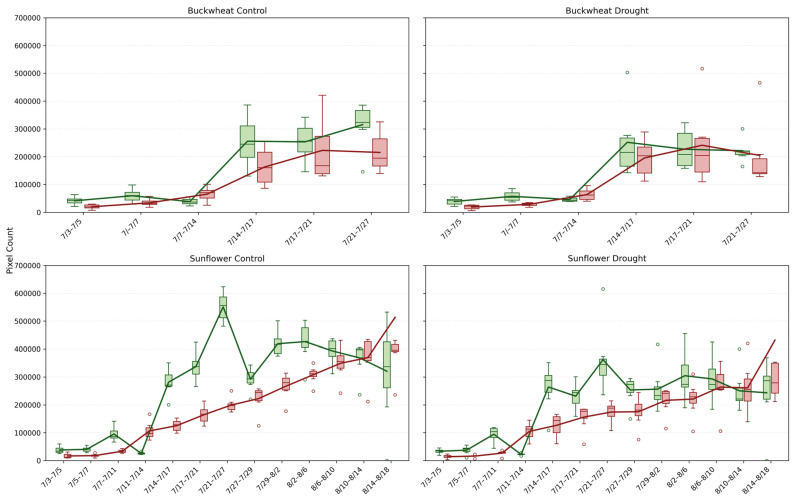
Box-and-whisker plots showing the distributions of positive change (Change^+^; green) and negative change (Change^−^; red) pixel counts for buckwheat (**top row**) and sunflower (**bottom row**) plants under control (**left column**) and drought (**right column**) treatments across their respective sampling intervals. Each box represents per-sample variability at each time point, and the overlaid lines show the corresponding mean values. Unfilled circles denote individual sample values plotted beyond the whiskers, indicating outliers relative to the interquartile range.

This study provides a detailed comparison of growth and decay trends in buckwheat and sunflower under control and drought conditions, illustrated in Figure 8. For both crops, growth and decay are quantified by tracking pixel counts over time, representing plant changes between consecutive time points. The box-and-whisker plots reveal not only the overall magnitude of growth and decay with averaging lines, but also the within-timepoint variability across biological replicates under the induced drought conditions on 14 July.

The Change^+^ and Change^−^ phenotypes reveal fundamental differences in how buckwheat and sunflower reorganize their canopies over time under both control and drought conditions. In buckwheat, both positive and negative changes reach large magnitudes after mid-July, frequently exceeding ~200,000 pixels, with comparable scales across treatments. The similar magnitude of gain and loss indicates that buckwheat growth is driven by rapid structural turnover, including leaf overlap, occlusion, and reorientation, rather than sustained net expansion. Under drought, this turnover is not suppressed; instead, variability increases, suggesting that water limitation amplifies architectural instability rather than constraining visible change. The wide distributions and frequent outliers highlight a highly heterogeneous response, in which individual plants adjust through sporadic, high-magnitude reconfigurations.

Sunflower exhibits a more coordinated temporal pattern. Under control conditions, Change^+^ reaches substantially higher values, often exceeding 400,000–500,000 pixels during peak growth, whereas Change^−^ remains lower, reflecting dominant canopy expansion with limited structural loss. Drought clearly constrains this process, reducing both Change^+^ and Change^−^, with peak positive change typically remaining below 300,000 pixels. This suppression indicates that water stress limits not only biomass accumulation but also the rate of structural reorganization, producing a more static and uniform canopy. Together, these patterns demonstrate that Change^+^ and Change^−^ distinguish species-specific growth strategies: buckwheat responds to stress through volatile reconfiguration, whereas sunflower exhibits coordinated expansion that is strongly attenuated under drought.

#### 4.2.2. Qualitative Analysis of Change ($Change^{+}$, $Change^{-}$)

**Figure 9:**
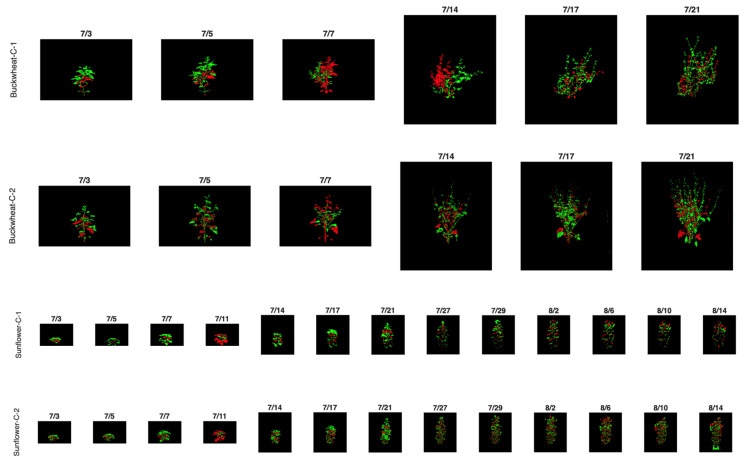
Qualitative visualization of positive (Change^+^; green) and negative (Change^−^; red) temporal changes for sunflower and buckwheat plants across sequential time points. Green regions indicate newly visible plant material, while red regions highlight pixels that disappeared due to occlusion, leaf repositioning, or structural loss.

In Figure 9, the qualitative Change^+^ (green) and Change^−^ (red) visualizations reveal how temporal change is spatially distributed across plant canopies and clarify the structural mechanisms underlying the quantitative trends. In buckwheat, both control and drought plants show dense intermixing of positive and negative change throughout the canopy at nearly all time points. Even early in development, change is fragmented and spatially dispersed, reflecting frequent small-scale leaf repositioning and overlap. As plants grow larger, particularly after mid-July, change becomes increasingly scattered across branches and leaf clusters rather than concentrated in specific regions. Under drought, this spatial fragmentation intensifies, with irregular patches of gain and loss appearing across the entire canopy, indicating that stress amplifies localized instability rather than driving coherent directional growth or decline.

Sunflower exhibits a markedly different spatial pattern. Change^+^ regions are more contiguous and tend to follow coherent growth fronts along the upper canopy and expanding leaf margins, while Change^−^ regions remain sparse and spatially constrained. This organization persists across replicates and over time, reflecting sunflower’s coordinated architectural development. Under drought, the spatial footprint of Change^+^ is visibly reduced, but its organization remains intact: growth occurs in fewer, more centralized regions rather than being redistributed erratically. The limited and structured appearance of Change^−^ further indicates that sunflower adjusts canopy size primarily by constraining expansion rather than through widespread reorientation or occlusion. Together, these spatial patterns reinforce the species-level distinction revealed by the quantitative phenotypes: buckwheat responds to stress through distributed, high-frequency structural reconfiguration, whereas sunflower maintains architectural coherence while modulating growth magnitude.

#### 4.2.3. Fragmentation of Change (N$Change^{+}$, N$Change^{-}$)

**Figure 10:**
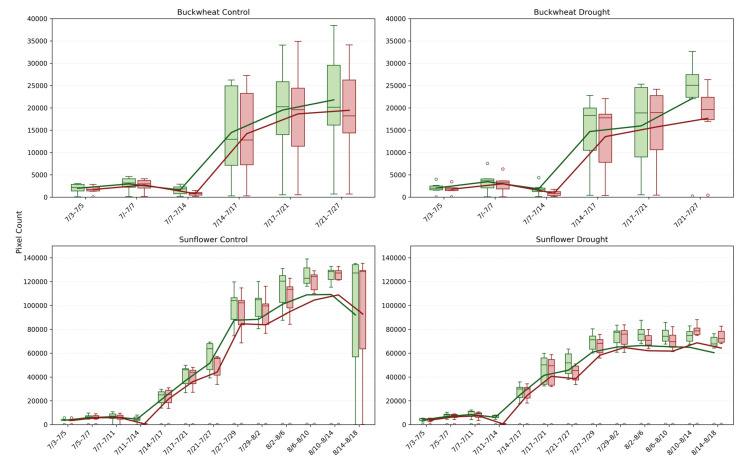
Temporal dynamics of normalized positive (NChange^+^, green) and negative (NChange^−^, red) pixel changes for buckwheat (**top row**) and sunflower (**bottom row**) under control (**left**) and drought (**right**) conditions. Each time interval shows the distribution of plant-level responses across all samples (boxplots), with overlaid mean trajectories for NChange^+^ and NChange^−^. Unfilled circles denote individual sample values plotted beyond the whiskers, indicating outliers relative to the interquartile range.

In Figure 10, the NChange^+^ and NChange^−^ phenotypes quantify the number of distinct structural change events between consecutive time points, revealing the degree of fragmentation or coordination in canopy dynamics over time. In buckwheat, both control and drought plants exhibit elevated and highly variable NChange values after mid-July, frequently reaching ~20,000–30,000 events. This high event count indicates that temporal change in buckwheat is distributed across many small, independent regions rather than driven by a few dominant structural shifts. Under drought, NChange distributions broaden further, with more extreme outliers and intermittent low-activity intervals, indicating that stress increases the irregularity of structural turnover rather than imposing a consistent suppression. These patterns reinforce buckwheat’s characterization as a species with fine-grained, high-frequency canopy reconfiguration, where growth and decay occur through numerous localized adjustments.

Sunflower exhibits a markedly different fragmentation profile. Under control conditions, NChange^+^ and NChange^−^ rise smoothly to peak values often exceeding ~100,000 events, reflecting coordinated but widespread structural change during rapid vegetative expansion. Despite the larger absolute counts, the distributions remain comparatively tight, indicating strong synchrony among individuals. Under drought, sunflower shows a clear reduction in NChange magnitude, typically peaking near ~60,000–80,000 events, along with reduced dispersion. This contraction indicates that water stress limits not only the magnitude of growth but also the number of independently changing regions, producing a more constrained and homogeneous canopy response. Together, these results demonstrate that NChange phenotypes capture an essential dimension of temporal behavior, whether change is fragmented or coordinated, indicating species-specific stress strategies that are invisible to magnitude-based or static phenotypes.

#### 4.2.4. Dominant Structural Events ($MaxChange^{+}$, $MaxChange^{-}$)

**Figure 11:**
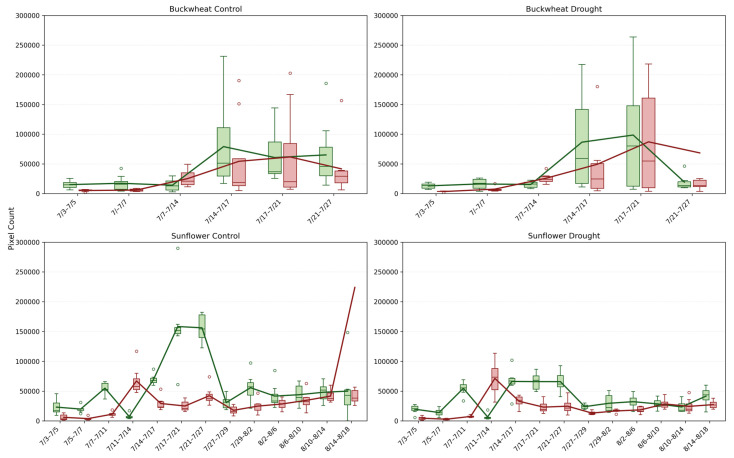
Maximum positive (MaxChange^+^; green) and maximum negative (MaxChange^−^; red) temporal change across buckwheat and sunflower plants under control and drought conditions. Each boxplot represents the largest contiguous region of newly visible plant material (MaxChange^+^) or material that became occluded or disappeared (MaxChange^−^) between consecutive imaging dates.

The MaxChange^+^ and MaxChange^−^ phenotypes (Figure 11) identify the largest contiguous structural change between consecutive imaging dates, revealing whether canopy dynamics are driven by dominant, canopy-scale events or by many smaller adjustments. In buckwheat, both control and drought plants show moderate but highly variable MaxChange values after mid-July, with peak events frequently reaching ~80,000–150,000 pixels. The intermittent nature of these peaks, coupled with broad distributions, indicates that buckwheat undergoes occasional large reorganization events embedded within an otherwise highly fragmented change process. Under drought, MaxChange^+^ and MaxChange^−^ magnitudes remain comparable to or slightly exceed control values in some intervals, suggesting that stress does not suppress dominant events but instead increases their unpredictability. This pattern reinforces the view of buckwheat as architecturally plastic, in which stress manifests as sporadic, large-scale repositioning rather than as sustained directional growth or decline.

Sunflower exhibits a more structured and contrasting MaxChange signature. Under control conditions, MaxChange^+^ shows pronounced peaks during early rapid growth, often exceeding ~150,000 pixels, reflecting coordinated expansion of large leaf surfaces and upper canopy regions. MaxChange^−^ remains lower and more stable, indicating limited large-scale structural loss. Under drought, both MaxChange^+^ and MaxChange^−^ are markedly attenuated, typically remaining below ~100,000 pixels and exhibiting narrower distributions. This reduction indicates that water stress constrains the amplitude of dominant structural events, leading to fewer large-scale reorganizations and a more static canopy. Together, these results show that MaxChange phenotypes capture a distinct temporal layer: buckwheat relies on irregular, stress-amplified dominant events within a volatile canopy, whereas sunflower expresses predictable, growth-driven dominant events that are strongly dampened under drought. This distinction cannot be inferred from cumulative growth or event counts alone, highlighting the value of MaxChange for identifying canopy-scale reorganization strategies.

#### 4.2.5. Spatial Organization of Change ($Dispersion^{+}$)

**Figure 12:**
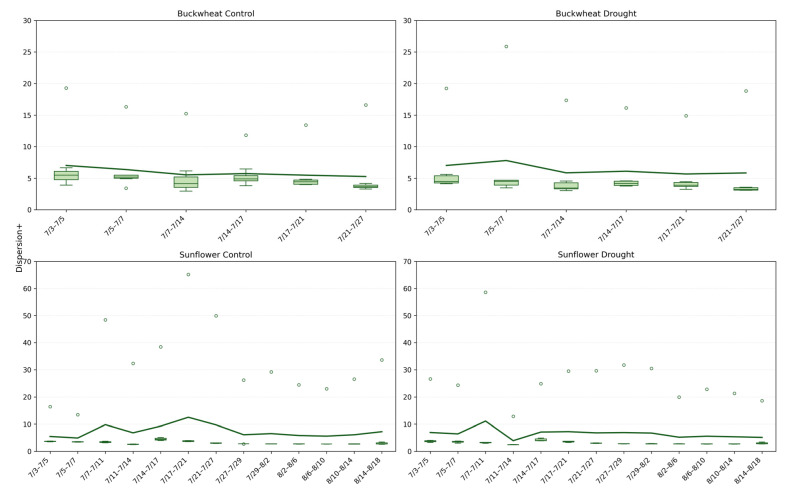
Dispersion^+^ phenotype for buckwheat (**top**) and sunflower (**bottom**) under control (**left**) and drought conditions (**right**). Boxplots show the spatial Dispersion^+^ of temporal changes, quantified as the spread of change pixels across each canopy, calculated between consecutive imaging dates. Higher values indicate that structural changes occurred across widely distributed canopy regions, whereas lower values reflect more localized transitions. The solid green line overlays the boxplots and represents the mean Dispersion^+^ value at each time point, highlighting temporal trends within each treatment and species.

The Dispersion^+^ phenotype (Figure 12) quantifies the spatial distribution of positive structural changes across the canopy, distinguishing localized reconfiguration from coordinated, canopy-wide adjustment. In buckwheat, Dispersion^+^ values remain consistently low under both control and drought conditions, typically clustering around ~4–6, indicating that structural changes are spatially concentrated rather than broadly distributed. This low Dispersion^+^ aligns with earlier findings of high fragmentation and turnover: buckwheat’s temporal dynamics are driven by many small, localized adjustments within specific leaf clusters rather than by synchronized changes across the canopy. Under drought, Dispersion^+^ increases only marginally and irregularly, suggesting that stress does not fundamentally alter the spatial organization of change but instead amplifies variability within already localized regions of activity. Sunflower exhibits a contrasting Dispersion^+^ profile. Under control conditions, Dispersion^+^ values are higher and more variable, frequently exceeding ~10 and occasionally reaching much higher values, reflecting coordinated structural adjustments spanning vast canopy regions during periods of rapid growth. This elevated Dispersion^+^ indicates that sunflower growth involves spatially coherent expansion, consistent with its broad leaves and unified architectural form. Under drought, Dispersion^+^ remains moderate but shows sporadic high outliers, suggesting that while overall growth and reorganization are constrained, occasional stress-induced adjustments can affect large portions of the canopy simultaneously. Together, these patterns demonstrate that Dispersion^+^ captures a spatial dimension of temporal dynamics that complements Change, NChange, and MaxChange: buckwheat adapts through localized, fine-scale rearrangements, whereas sunflower relies on broader, canopy-level coordination that is selectively disrupted under stress.

#### 4.2.6. Statistical Analysis of Unidimensional Temporal Phenotypes

The statistical comparisons in Table 3 evaluate within-species control versus drought contrasts using plant-level, temporally aggregated phenotypes. In buckwheat, none of the evaluated temporal phenotypes show statistically significant differences between control and drought treatments (all *p* ≥ 0.27). This lack of population-level significance supports the interpretation that buckwheat’s response to drought is characterized by high inter-plant variability and irregular structural turnover, rather than a consistent directional shift in canopy dynamics. Although individual buckwheat plants frequently undergo large structural changes, these responses do not converge into a coherent stress signature detectable at the population scale.

In contrast, sunflower exhibits clear and selective drought sensitivity in specific temporal phenotypes. Both Change^+^ and MaxChange^+^ show strong and statistically significant differences between control and drought conditions (*p* < 0.001), indicating that drought reliably suppresses the magnitude of dominant positive structural change events. These results suggest that sunflower’s canopy dynamics under water limitation are constrained primarily through reductions in large-scale growth or expansion events, rather than through broad suppression of all temporal activity. The Change^−^, NChange^+^, NChange^−^, and Dispersion^+^ phenotype do not reach statistical significance (*p* ≥ 0.16), indicating that drought does not uniformly affect fragmentation, decay, or spatial Dispersion^+^ of change at the population level.

Together, these findings highlight species-specific modes of drought response: buckwheat exhibits diffuse, heterogeneous temporal behavior that resists population-level detection, whereas sunflower shows coordinated and directional suppression of dominant growth dynamics. The selective sensitivity of Change^+^ and MaxChange^+^ in sunflower demonstrates that the proposed temporal phenotypes capture biologically meaningful aspects of canopy regulation under stress that are not apparent from static or single-time-point measurements.

### 4.3. Evaluation of Proposed Unidimensional Perspective Phenotypes

We focus our study on buckwheat and sunflower and analyzed using the three UDP phenotype measurements extracted from the images: TAR_min_, TAR_max_ and TWR. We used our concurrent imaging approach to analyze the phenotypes’ ability to detect structural variations that correspond to stress responses.

#### 4.3.1. Temporal Dynamics of Perspective Ratios (TARmin, TARmax, and TWR)

**Figure 13:**
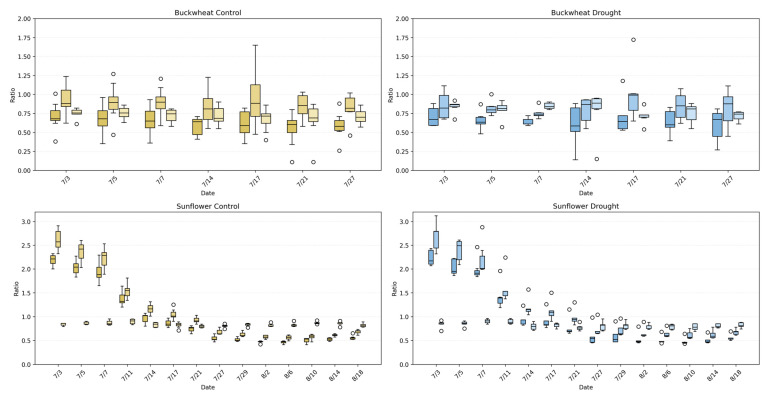
UDP phenotypes (TAR_min_, TAR_max_, and TWR) for buckwheat (**top**) and sunflower (**bottom**) under control (**left**) and drought (**right**) conditions. Boxplots show the distribution of ratio values across plants at each imaging date, capturing changes in the vertical–horizontal geometry of the canopy over time. Each time point has 3 different boxplots with 3 varying shades; the left box (darkest) represents TAR_min_, the middle box (mid-tone) represents TAR_max_, and the left box (lightest) represents TWR. Higher values reflect elongated or vertically dominant presentations, while lower values represent wider or more compact structures. Unfilled circles denote individual sample values plotted beyond the whiskers, indicating outliers relative to the interquartile range.

The perspective phenotypes (TARmin, TARmax, and TWR) in Figure 13 characterize global canopy geometry by capturing how plant silhouettes vary across viewpoints, revealing architectural coordination rather than temporal change. In buckwheat, all three ratios remain confined to a narrow range across time and treatments, typically centered between ~0.6 and ~0.9. The substantial overlap between control and drought distributions indicates that water limitation does not induce a consistent shift in buckwheat’s global geometric proportions. Instead, the variability observed across dates reflects small, localized stem and leaf reorientations that alter individual views without producing systematic asymmetry or directional elongation. This stability across perspective metrics reinforces earlier findings that buckwheat adapts through fine-scale, localized adjustments rather than coordinated architectural reconfiguration.

Sunflower exhibits a markedly different geometric signature. Under control conditions, TAR_max_ and TAR_min_ reach substantially higher values early in development, often exceeding ~2.0–2.5, reflecting strong vertical dominance and coordinated canopy elongation across perspectives. Over time, these ratios decline toward values near ~0.7–1.0 as the canopy broadens and stabilizes. Under drought, sunflower maintains the same general geometric trajectory but with increased Dispersion^+^ and more frequent deviations from the control distributions, particularly during early and mid-growth stages. This increased variability indicates reduced architectural coordination under stress, where vertical elongation and lateral expansion become less synchronized across viewpoints. Together, these results demonstrate that perspective phenotypes effectively distinguish species-level architectural strategies and reveal how drought disrupts global geometric coherence in sunflower while leaving buckwheat’s already flexible structure largely unchanged.

#### 4.3.2. Aggregated Geometric Distributions

**Figure 14:**
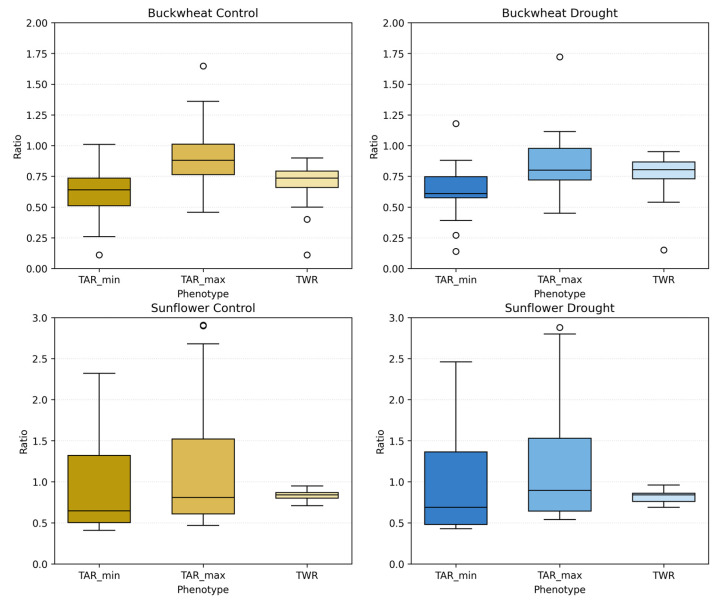
Aggregate distributions of perspective-based phenotypes: TAR_min_, TAR_max_, and TWR, for buckwheat (**top row**) and sunflower (**bottom row**) under control (**left**) and drought (**right**) conditions. Each boxplot summarizes all imaging dates for the corresponding treatment. Unfilled circles denote individual sample values plotted beyond the whiskers, indicating outliers relative to the interquartile range.

The aggregated distributions of TAR_min_, TAR_max_, and TWR in Figure 14 reveal clear and persistent species-level differences in global canopy geometry, which are only weakly modulated by drought. Buckwheat exhibits compact and tightly clustered ratio values across all three phenotypes, with medians generally confined to ~0.6–0.9. The narrow interquartile ranges and substantial overlap between control and drought treatments indicate that buckwheat maintains stable global proportions regardless of water availability. Even under drought, shifts in TAR_min_, TAR_max_, and TWR remain modest, reinforcing that buckwheat’s architectural adjustments occur through localized reorientation rather than through systematic changes in overall canopy shape.

Sunflower exhibits substantially broader and more variable distributions, particularly for TAR_min_ and TAR_max_, reflecting its inherently dynamic and vertically dominant architecture. Under control conditions, these ratios span a wide range and frequently exceed 2.0, indicating substantial elongation and pronounced directional structure. Under drought, the distributions broaden further and show increased dispersion, suggesting reduced geometric coordination and greater heterogeneity in how canopies present across viewpoints. In contrast, TWR remains comparatively stable across treatments, indicating that drought affects vertical–horizontal balance more strongly than proportional lateral spread. Together, these aggregated results confirm that UDP phenotypes robustly distinguish species-level architectural identity and capture drought-induced geometric irregularity in sunflower, while buckwheat remains geometrically stable despite stress.

#### 4.3.3. Statistical Analysis of Unidimensional Perspective Phenotypes

The statistical comparisons in Table 4 indicate that the UDP show no statistically significant control–drought differences when evaluated using plant-level aggregated values for either species. In buckwheat, TAR_max_ and TAR_min_ remain stable under drought (*p* = 0.63 and *p* = 0.73), and TWR likewise shows no population-level shift (*p* = 0.19), suggesting that water limitation does not induce a consistent change in global canopy proportions when responses are averaged across the season. This outcome is consistent with a heterogeneous, non-directional response in which individual plants may fluctuate substantially, but the population does not converge toward a unified geometric signature.

Similarly, sunflower exhibits no significant differences between control and drought TAR_max_, TAR_min_, and TWR (*p* = 0.54, *p* = 0.64, and *p* = 0.11, respectively), indicating that drought does not produce a uniform shift in overall aspect ratio or width–height balance at the population level after temporal aggregation. Although TWR shows the strongest trend in sunflower (*p* = 0.11), it does not reach significance, reinforcing that UDP descriptors are relatively insensitive to drought effects when time-dependent structure is collapsed.

Importantly, this aggregation behavior contrasts with the UDT phenotypes, which retain drought sensitivity under the same plant-level statistical framework. Unlike UDP metrics, UDT phenotypes encode the timing, magnitude, and frequency of discrete structural change events, allowing coordinated stress responses to remain detectable at the population scale. Together, these results support the interpretation that UDP phenotypes primarily characterize coarse canopy geometry, whereas UDT phenotypes capture dynamic, stress-responsive growth behavior that is not recoverable from global proportions alone.

### 4.4. Evaluation of Proposed Unidimensional Modality Phenotypes

We focus our study on buckwheat and sunflower and analyzed using the two UDM phenotype measurements extracted from the images: Intermodal Correlation (IC) and Intermodal Mutual Information (IMI).

#### 4.4.1. Intermodal Dependency Across Species and Treatment (IC and IMI)

**Figure 15:**
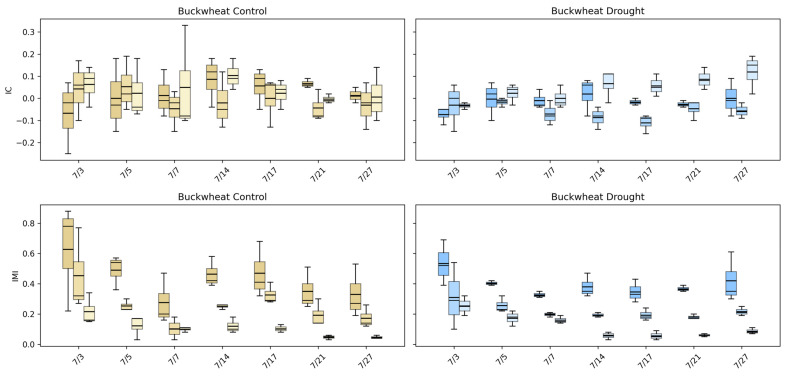
Intermodal correlation (IC) and intermodal mutual information (IMI) phenotypes for buckwheat (**top**) and sunflower (**bottom**) under control (**left**) and drought (**right**) conditions. Each boxplot shows the distribution of IC or IMI values for three modality pairings: Visible–Fluorescence, Visible–Infrared, and Infrared–Fluorescence, colored in gold (control) and blue (drought).

The intermodal correlation (IC) and intermodal mutual information (IMI) phenotypes (Figure 15) quantify how structural, thermal, and biochemical signals co-vary across the canopy, providing a relational view of plant function that single-modality summaries cannot capture. In buckwheat, IC values remain centered near zero across all modality pairings and time points under both control and drought conditions, indicating weak and inconsistent linear relationships among visible, infrared, and fluorescence signals. This lack of strong correlation reflects buckwheat’s fragmented canopy structure, where local reorientation and occlusion decouple structural appearance from thermal and fluorescence responses. IMI values are higher than IC early in development, often exceeding ~0.4–0.6, but decline steadily through the season, suggesting that early growth stages exhibit some shared multimodal information that dissipates as the canopy becomes more complex and heterogeneous. It is worth noting that drought does not induce a consistent shift in either IC or IMI, reinforcing that buckwheat’s stress response is dominated by localized variability rather than coordinated physiological change across modalities.

Sunflower exhibits a more structured multimodal response. Under control conditions, IMI values are substantially higher early in development, often reaching ~0.5–0.7, indicating strong cross-information among visible, infrared, and fluorescence signals during rapid canopy expansion. As growth stabilizes, IMI declines, reflecting reduced cross-model coupling among modalities as structural and physiological processes decouple over time. IC values show modest but interpretable structure, with early-season deviations from zero that converge toward weak dependence later in development. Under drought, both IC and IMI distributions shift downward and become more compact, particularly for modality pairings involving infrared, indicating reduced coordination between structural growth and thermal or fluorescence responses. This stress-induced reduction in multimodal dependency suggests a breakdown in the synchrony between physical architecture and physiological function. Together, these results demonstrate that UDM phenotypes capture physiologically meaningful stress signatures in sunflower that are invisible to single-modality analyses, while confirming that buckwheat’s multimodal behavior remains weakly coupled and largely insensitive to drought at the population level.

#### 4.4.2. Temporal Evolution of Intermodal Relationships

**Figure 16:**
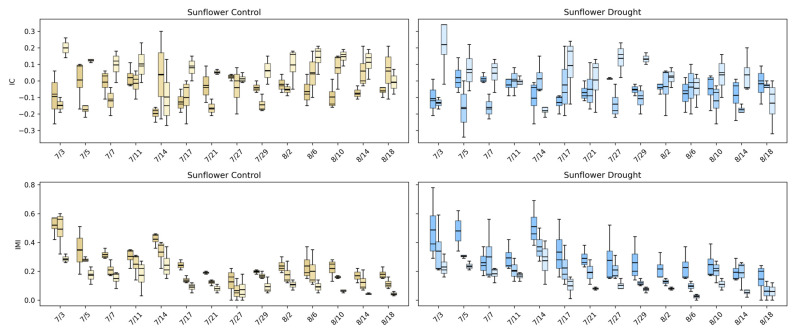
Intermodal correlation (IC) and intermodal mutual information (IMI) for sunflower under control (**left**) and drought (**right**) conditions across three modality pairings: Visible–Fluorescence, Visible–Infrared, and Infrared–Fluorescence. Boxplots show the distribution of IC and IMI values for each imaging date in the control (gold) and drought (blue) treatments. IC measures linear dependence among modalities, while IMI captures nonlinear mutual dependence.

Figure 16 highlights how intermodal relationships in sunflower evolve and how drought alters these dynamics. Under control conditions, IC values for visible–fluorescence and visible–infrared pairings fluctuate around mildly positive and negative values early in development, reflecting active coordination between structural growth and physiological processes during rapid canopy expansion. As the canopy matures, IC values converge toward zero, indicating a gradual decoupling of linear relationships among modalities as growth stabilizes. This transition suggests that early sunflower development is characterized by synchronized structural and physiological activity, which becomes less tightly linked once the canopy reaches architectural maturity.

IMI reveals a stronger and more interpretable drought signal. Under control conditions, IMI values are highest early in the season, frequently exceeding ~0.4–0.6, indicating substantial shared information across visible, infrared, and fluorescence modalities during vigorous growth. These values decline steadily over time, reflecting a reduction in multimodal dependence as developmental processes slow. Under drought, IMI values are consistently lower at nearly all time points and exhibit reduced dispersion, indicating that water stress limits not only growth but also the complexity and richness of cross-modal interactions. Together, the downward shift in IC and the sustained reduction in IMI demonstrate that drought disrupts the normal synchrony between sunflower’s structural and physiological signals. These results confirm that modality-based phenotypes capture stress-induced decoupling processes that are invisible to single-modality or purely structural analyses, providing a critical physiological dimension to concurrent image-based phenotyping.

#### 4.4.3. Statistical Analysis of Unidimensional Modality Phenotypes

The statistical comparisons in Table 5 indicate that the UDM phenotypes do not exhibit statistically significant control–drought differences when evaluated using plant-level aggregated values in either species (all *p* ≥ 0.18). In buckwheat, none of the IC or IMI modality pairings (Visible–Fluorescence, Visible–Infrared, or Infrared–Fluorescence) show evidence of a consistent population-wide shift under drought, suggesting that water stress does not impose a uniform reorganization of multimodal coupling across the growing season. Instead, buckwheat’s multimodal behavior remains weakly coordinated and highly variable, consistent with its diffuse and irregular structural response observed in earlier analyses.

Similarly, in sunflower, no IC or IMI phenotype reaches statistical significance for the control–drought contrast (all *p* ≥ 0.18), despite pronounced temporal changes in individual modality signals. This indicates that drought effects in sunflower primarily manifest as time-dependent alterations in multimodal relationships, rather than as constant offsets that persist when responses are averaged across the full season. As a result, plant-level aggregation attenuates transient periods of strong coupling or decoupling between modalities, yielding non-significant population-level statistics.

Notably, IC and IMI phenotypes also yield nearly identical control–drought statistics within each species, reflecting the fact that linear and nonlinear measures of multimodal dependence track the same underlying coordination structure when temporal information is collapsed. This behavior contrasts sharply with that of the UDT phenotypes, which retain sensitivity to drought after aggregation because they explicitly encode the timing, magnitude, and frequency of discrete structural change events. Together, these results indicate that UDM phenotypes are effective descriptors of multimodal organization over time, but, like UDP and TAR-based metrics, lose discriminatory power under temporal averaging. This further highlights the importance of event-based temporal phenotyping for detecting coordinated stress responses at the population level.

## 5. Discussion

Throughout this study, unidimensional phenotyping refers to isolating variation along a single phenotyping axis using concurrent images, rather than fusing information across time, modality, and viewpoint simultaneously. This design choice prioritizes interpretability and mechanistic attribution of observed stress responses. The results of this study demonstrate the value of unidimensional temporality, perspective, and modality phenotypes for capturing plant responses to abiotic stress beyond what is achievable with traditional single-image or cumulative descriptors.

Physiological interpretation of the proposed phenotypes must be understood in terms of indirect manifestation rather than direct measurement. Drought stress alters plant water status, stomatal regulation, and photosynthetic efficiency, which in turn influence leaf orientation, canopy density, thermal dissipation, and fluorescence emission. The unidimensional temporality phenotypes capture how these physiological processes express themselves as spatially fragmented or coordinated structural change over time, while the modality phenotypes quantify whether structural, thermal, and biochemical signals remain synchronized or become decoupled. Consequently, the proposed phenotypes are best interpreted as structural–physiological proxies that bridge imaging observables with underlying biological regulation, rather than as substitutes for direct physiological assays.

### 5.1. Phenotypic Capability Differences Between Conventional and Unidimensional Analytics

Prior phenotyping frameworks derived largely from visible imagery have emphasized projected area, aspect ratio, and other holistic traits [8,17], which successfully capture coarse architectural patterns but provide limited insight into spatially localized growth, occlusion, and physiological decoupling. By isolating and intensively exploiting concurrency within individual phenotyping dimensions, the proposed taxonomy reveals complementary and biologically interpretable stress signatures that are consistent with, yet extend beyond, existing literature. Selected biological differences are referenced in Table 6.

### 5.2. Biological Interpretation of Unidimensional Phenotypes

UDT phenotypes, including Change^+^, Change^−^, NChange^+^/^−^, MaxChange^+^/^−^, and Dispersion^+^, revealed species-specific growth strategies that are not captured by projected area alone. Previous temporal studies have reported drought-induced reductions in canopy expansion for maize and sunflower using cumulative traits such as area or height [8,17], a pattern corroborated here for sunflower, which showed coordinated suppression across Change^+^, NChange^+^, NChange^−^, and MaxChange^+^. In contrast, buckwheat exhibited large but statistically non-significant drought effects characterized by fragmented, high-frequency turnover, supporting observations in fine-canopy species that stress responses are often expressed through localized reorientation rather than uniform biomass loss. This contrast demonstrates that UDT phenotypes quantify whether drought manifests as systematic growth suppression or as stochastic architectural reconfiguration. Biologically, this distinction is consistent with early-stage drought accommodation through architectural plasticity versus intermediate-stage stress characterized by coordinated suppression of expansion. In fine-canopy species such as buckwheat, drought responses may be absorbed through localized reorientation and overlap without immediate biomass reduction, whereas broader-leaf species such as sunflower tend to express stress through synchronized canopy-scale growth inhibition. UDT phenotypes therefore provide interpretable indicators of how drought alters growth dynamics and structural turnover beyond net biomass change.

UDP phenotypes extended classical aspect-ratio–based metrics by leveraging multi-view concurrency to better approximate true canopy geometry. Prior perspective-based phenotypes assume approximate rotational symmetry or rely on single-view silhouettes [8,17], which limits sensitivity to asymmetric reorganization. Our results show that buckwheat maintains stable TAR_min_ and TAR_max_ under drought, with only TWR shifting, indicating subtle lateral restructuring, whereas sunflower exhibits large intrinsic geometric variability with weak treatment effects when aggregated. These findings contrast with earlier reports that rely on single-view aspect ratios to infer stress-induced elongation or compaction, illustrating that UDP phenotypes are better suited to disentangle species-level architectural identity from viewpoint-driven artifacts. From a biological perspective, viewpoint-dependent asymmetry reflects species-specific architectural strategies that influence light interception, self-shading, and mechanical stability under stress. Stable TAR_min_ and TAR_max_ values in buckwheat suggest preservation of a flexible, laterally adaptive canopy capable of redistributing tissue without large-scale geometric collapse, whereas the higher intrinsic variability observed in sunflower indicates a morphology less amenable to asymmetric reconfiguration. UDP phenotypes therefore provide a biologically grounded description of how drought alters canopy organization across viewpoints rather than merely its projected shape from a single perspective.

UDM phenotypes provided a physiological dimension by quantifying cross-modal dependencies using intermodal correlation (IC) and mutual information (IMI). Previous multimodal phenotyping efforts have largely treated modalities independently or combined them through simple indices [6,7], limiting interpretation of how thermal, structural, and biochemical signals interact. In this study, buckwheat exhibited weak and drought-insensitive modality coupling, except for a significant Visible–Infrared IC shift, whereas sunflower showed strong early-season multimodal coherence that declined over time, consistent with reports of stress-induced decoupling between photosynthetic efficiency and canopy structure in drought-sensitive species. The robust cross-species differences in Visible–Fluorescence IC and IMI further support literature suggesting that multimodal relationships encode fundamental architectural and physiological traits not observable within any single imaging channel. Biologically, reductions in intermodal dependency are consistent with progressive loss of coordination between structural growth, thermal regulation, and biochemical activity during drought progression. Strong early-season multimodal coherence in sunflower suggests tightly synchronized physiological and structural regulation under favorable conditions, whereas the subsequent decline under drought reflects stress-induced decoupling rather than abrupt failure of individual processes. UDM phenotypes therefore offer an interpretable relational view of drought response that complements single-modality physiological proxies without claiming direct measurement of underlying physiological rates.

### 5.3. Broader Implications and Scalability

The findings of this study are strongly supported by and extend prior work in high-throughput phenotyping and multimodal plant analytics. Earlier taxonomies by Choudhury et al. [17] and the subsequent maize-focused framework [27] demonstrated that structural, physiological, and temporal phenotypes derived from single images or sequential processing could capture coarse architectural traits, yet they were limited in their ability to describe spatially localized growth, occlusion, and stress-induced decoupling. Our unidimensional phenotypes operationalize this gap by explicitly leveraging concurrent image analysis, a principle motivated by advances in cosegmentation methodologies such as OSC-CO^2^ [7] and multi-feature repositories like CosegPP [6], which showed that simultaneous processing of related images improves segmentation accuracy by up to 45%. By embedding these principles directly into phenotype definitions, rather than treating them solely as preprocessing steps, we demonstrate that dynamic growth turnover (UDT), viewpoint-aware structural organization (UDP), and cross-modal physiological coupling (UDM) reveal biologically meaningful patterns that are not detectable with conventional single-image or cumulative metrics. The consistency of sunflower drought signatures across Change^+^, NChange^+^, MaxChange^+^, Dispersion^+^, and intermodal IMI metrics provides empirical validation of the manuscript’s central thesis: that systematically exploiting concurrency within a single phenotyping dimension yields interpretable and transferable descriptors of stress response.

Looking forward, the implications of this framework extend beyond controlled-environment phenotyping. Remote sensing platforms such as OpenET, which integrate multisensor satellite data to estimate evapotranspiration at field scales [54], could benefit from cosegmentation-driven analytics to improve field boundary delineation, cloud masking, and evapotranspiration diagnosis through improved multimodal coherence. Embedding unidimensional concurrent phenotypes into these large-scale monitoring systems represents a promising pathway for translating high-throughput phenomics into operational water-resource and crop-stress decision support, bridging greenhouse-scale image analytics with regional-scale remote sensing infrastructures.

### 5.4. Translational Use Cases and Operational Integration

The proposed unidimensional phenotypes are designed to function as intermediate analytics that can be embedded into breeding pipelines, early stress diagnosis, and agricultural decision support systems, rather than as standalone decision rules. Below, we outline concrete example use cases illustrating how these phenotypes can be operationalized.

#### 5.4.1. Use Case 1: Early Stress Diagnosis (Greenhouse or Phenotyping Facility)

Event-based temporal phenotypes such as Change^+^, MaxChange^+^, and Dispersion^+^ can be used to detect early deviations from normal growth dynamics before cumulative traits (e.g., projected area) diverge. For example, a sustained reduction in MaxChange^+^ combined with increased fragmentation (NChange^+/−^) relative to control baselines may indicate early stress-induced suppression of coordinated expansion. In practice, thresholds would be defined relative to species- and stage-specific control distributions (e.g., percentile-based deviation from historical controls), enabling early stress flagging without requiring fixed universal cutoffs.

#### 5.4.2. Use Case 2: Breeding and Genotype Ranking

In breeding trials, genotypes can be ranked based on their unidimensional phenotype profiles rather than absolute growth values. For instance, drought-tolerant candidates may be characterized by preserved MaxChange^+^ magnitude, lower Dispersion^+^ of change, or delayed loss of intermodal dependency under stress. These phenotypes provide interpretable descriptors of stress adaptation strategies, enabling breeders to distinguish genotypes that maintain coordinated growth from those that rely on fragmented or compensatory reorganization. Thresholds in this context are comparative rather than absolute, derived from rank-ordering or clustering across genotypes within the same experimental design.

#### 5.4.3. Use Case 3: Decision Support and Monitoring Systems

In decision support systems, unidimensional phenotypes can be integrated as time-series features feeding higher-level classifiers or rule-based alerts. For example, a rolling-window decrease in intermodal mutual information (IMI) between visible and infrared modalities could serve as an indicator of decoupling between structural growth and thermal regulation, prompting irrigation review or targeted inspection. Rather than fixed thresholds, adaptive thresholds can be learned from historical season-level data, allowing alerts to be triggered based on deviations from expected trajectories rather than absolute phenotype values.

## 6. Conclusions

This study introduced a set of unidimensional phenotypes that leverage temporality, perspective, and it does not aim to quantify the physiological accuracy of drought stress responses. Instead, it evaluates whether the proposed unidimensional phenotypes exhibit sufficient statistical discriminative power to distinguish between control and drought treatments using image-based data alone. By analyzing growth dynamics, structural adaptations, and physiological relationships across multiple imaging channels, these phenotypes provide a more holistic understanding of plant behavior and adaptation compared to traditional, static phenotyping approaches demonstrating their methodological transferability across species and stress conditions, with empirical validation provided for two morphologically distinct broadleaf species. Through concurrent imaging and advanced computational analyses, we demonstrated how these phenotypes offer deeper insights into the interrelationships between different plant health metrics and their responses over time, distinguishing themselves from conventional single-image methods. Our proposed taxonomy, supported by the newly developed SIMID and SIPID datasets, serves as a foundation for further advancing plant phenotyping and enhancing our ability to evaluate diverse crops with varying architectural features under controlled conditions.

This study demonstrates that systematically exploiting concurrent images within individual temporal, modal, and perspective dimensions enables the extraction of phenotypes that reveal plant growth dynamics, structural organization, and multimodal physiological relationships that are not captured by conventional single-image or cumulative approaches. The proposed unidimensional phenotypes were shown to distinguish species-specific architectural strategies and to identify selective drought-induced disruptions in canopy development and intermodal coupling. Together, these findings confirm that even conceptually simple metrics, when derived from concurrent image analysis, can provide biologically meaningful and transferable insight into plant stress response mechanisms.

Limitations exist in the current study, including the computational complexity of multi-dimensional data processing and the potential for data variability across different species or environmental conditions. Additionally, the unidimensional phenotypes developed here, while informative, represent only a subset of the richer multi-view, multi-temporal, and cross-modal data available, indicating opportunities to extend these traits into higher-dimensional representations. Demonstrating reliable differentiation is a necessary first step in phenotype development because phenotypes that cannot discriminate treatments cannot subsequently be mapped to physiological processes. Accordingly, no destructive or direct physiological measurements (e.g., leaf water potential, stomatal conductance, or chlorophyll assays) were collected, and the results should be interpreted as evidence of discriminative sensitivity rather than physiological validation.

The present evaluation is limited to two dicot species with contrasting canopy architectures (buckwheat and sunflower). While the proposed unidimensional phenotypes are defined at the image-analysis level and are therefore architecture-agnostic in formulation, their biological interpretation may vary across plant forms. For example, monocot species with erect, vertically oriented leaves may express drought stress predominantly through leaf angle changes rather than canopy fragmentation, potentially emphasizing UDP phenotypes over UDT fragmentation metrics. Similarly, rosette architectures may exhibit reduced perspective asymmetry but heightened localized turnover. Explicit validation across monocots, rosette species, and woody seedlings remains an important direction for future work.

Future work will focus on integrating destructive ground-truth measurements to establish quantitative biological correspondence is a key direction for future work, refining the proposed phenotypes by incorporating machine learning techniques to enhance scalability and integrating additional dimensions of plant data to further improve phenotypic accuracy and biochemical analysis. Expanding these phenotypes to field-scale datasets, multiview reconstructions, and predictive modeling frameworks will help establish a standardized and extensible pipeline for next-generation plant phenotyping research. Continued research on optimizing computational models and exploring broader applications in diverse crop environments will ensure the robustness and versatility of these phenotyping approaches for improved agricultural productivity and stress resilience.

## Figures and Tables

**Table 1:** Overview of existing plant phenotyping datasets, detailing their temporal coverage, image views, modalities used, species studied, and environmental conditions. Modality abbreviations: Vis = visible light, IR = infrared, Fl = fluorescence, Vis-D = visible–depth.

Dataset	Temporality (Days)	Image Views	Modality	Species	Environmental Conditions
Panicoid-Phenomap-1 [9]	27	2 side views	Vis	Maize	Control
UNL-CPPD [17]	13	2 side views	Vis	Maize	Control
UNL-3DPPD [27]	13	10 side views	Vis	Maize	Control
FlowerPheno Dataset [44]	26	8 side views	Vis	Maize	Control
CosegPP [6]	7 to 14	4 side views	Vis, IR, Fl	Buckwheat, Sunflower	Control, Drought Stress
Leaf Segmentation Challenge [45]	30	Top view only	Vis	Arabidopsis, Tobacco	Control
Michigan State University Plant Imagery [46]	5 to 9	Top view only	Fl, IR, Vis, Vis-D	Arabidopsis, Bean	Control
Komatsuna Dataset [47]	10	Top view only	Vis, Vis-D	Komatsuna	Control

**Table 2:** Dataset summary of the SIMID and SIPID datasets for single dimension phenotypes. Modality abbreviations: Vis = visible light, IR = infrared, Fl = fluorescence.

Dataset	Temporality (Days) per Plant	Side View Perspective	Modality	Species (S)	Number of Biological Replicates per S	Environmental Condition (EC)	Number of Samples per EC
SIPID	7 to 14	0, 72, 144, 216	Vis–binary images	Buckwheat, Sunflower	14	Control, Drought	6 to 8
SIMID	7 to 15	0	Vis, IR, Fl	Buckwheat, Sunflower	6	Control, Drought	3

**Table 3:** Statistical comparisons of temporal phenotypes between control and drought treatments within each species. For each UDT phenotype, Welch’s two-sample *t*-tests were performed using plant-level aggregated values (mean across time per plant) as independent biological replicates. Reported values include the t-statistic and corresponding *p*-value for each comparison.

	Buckwheat	Sunflower	Buckwheat	Sunflower
			Control vs. Drought	Control vs. Drought
**Change^+^**	t = 1.16, *p* = 0.27	t = 4.26, *p* = 0.00	t = 1.16, *p* = 0.27	t = 4.26, *p* = 0.00
**Change^−^**	t = −0.23, *p* = 0.83	t = 1.54, *p* = 0.16	t = −0.23, *p* = 0.83	t = 1.54, *p* = 0.16
**Dispersion^+^**	t = −0.17, *p* = 0.87	t = 1.16, *p* = 0.88	t = −0.17, *p* = 0.87	t = 1.16, *p* = 0.88
**MaxChange^+^**	t = 0.03, *p* = 0.98	t = 6.02, *p* = 0.00	t = 0.03, *p* = 0.98	t = 6.02, *p* = 0.00
**MaxChange^−^**	t = −0.54, *p* = 0.61	t = 1.42, *p* = 0.20	t = −0.54, *p* = 0.61	t = 1.42, *p* = 0.20
**NChange^+^**	t = 0.12, *p* = 0.90	t = 1.29, *p* = 0.22	t = 0.12, *p* = 0.90	t = 1.29, *p* = 0.22
**NChange^−^**	t = 0.30, *p* = 0.77	t = 1.27, *p* = 0.23	t = 0.30, *p* = 0.77	t = 1.27, *p* = 0.23

**Table 4:** Statistical comparisons of UDP phenotypes, including TAR_max_, TAR_min_ and TWR. Welch’s two-sample *t*-tests were performed using plant-level aggregated values (mean across time per plant) to compare control versus drought treatments within each species and to assess cross-species differences. Reported values include the t-statistic and corresponding *p*-value. Significant results (*p* < 0.05, bolded) indicate phenotypes that reliably distinguish species-level architectural differences.

	Buckwheat	Sunflower	Buckwheat	Sunflower
			Control vs. Drought	Control vs. Drought
TAR_max_,	t = 0.49, *p* = 0.63	t = −0.64, *p* = 0.54	t = 0.49, *p* = 0.63	t = −0.64, *p* = 0.54
TAR_min_	t = −0.35, *p* = 0.73	t = −0.48, *p* = 0.64	t = −0.35, *p* = 0.73	t = −0.48, *p* = 0.64
TWR	t = −1.41, *p* = 0.19	t = 1.76, *p* = 0.11	t = −1.41, *p* = 0.19	t = 1.76, *p* = 0.11

**Table 5:** Statistical comparisons of UDM phenotypes, for buckwheat and sunflower under control and drought conditions. Welch’s two-sample *t*-tests were performed using plant-level aggregated values (mean across time per plant) to compare control versus drought treatments within each species. Reported values include the t-statistic and corresponding *p*-value. vf = visible-fluorescence, vi = visible-infrared, if = infrared-fluorescence.

	Buckwheat	Sunflower	Buckwheat	Sunflower
			Control vs. Drought	Control vs. Drought
**IC_vf**	t = 1.26, *p* = 0.30	t = −0.12, *p* = 0.91	t = 1.26, *p* = 0.30	t = −0.12, *p* = 0.91
**IC_vi**	t = 0.97, *p* = 0.43	t = 1.83, *p* = 0.18	t = 0.97, *p* = 0.43	t = 1.83, *p* = 0.18
**IC_if**	t = −0.23, *p* = 0.84	t = 1.50, *p* = 0.24	t = −0.23, *p* = 0.84	t = 1.50, *p* = 0.24
**IMI_vf**	t = 1.39, *p* = 0.73	t = −0.51, *p* = 0.65	t = 1.39, *p* = 0.73	t = −0.51, *p* = 0.65
**IMI_vi**	t = 0.97, *p* = 0.40	t = −0.19, *p* = 0.86	t = 0.97, *p* = 0.40	t = −0.19, *p* = 0.86
**IMI_if**	t = −0.63, *p* = 0.58	t = −0.30, *p* = 0.79	t = −0.63, *p* = 0.58	t = −0.30, *p* = 0.79

**Table 6:** Comparison of biological information captured by conventional HTP-derived phenotypes and proposed unidimensional phenotypes for drought-stress analysis.

Biological Process/Stress Manifestation	Conventional Phenotypes	Proposed Unidimensional Phenotypes
Net biomass accumulation/canopy expansion	Projected shoot/rosette area, growth/expansion rate [49,50]	Not measurable
Net growth suppression under drought	Relative growth/expansion rate [50,51]	Not measurable
Leaf senescence/chlorosis (visible symptom)	Color/greenness indices [52]	Not measurable unless you add color-based change masks
Global canopy openness/density (static state)	Compactness, hull ratio, projected area/convex-hull area [52,53]	Not measurable
Dynamic spatial fragmentation and turnover of canopy tissue	Not directly quantified by standard scalar traits; inferred only indirectly by change in compactness or area over time	NChange^+^/NChange^−^, Dispersion^+^, MaxChange^+^/MaxChange^−^
Leaf reorientation/wilting posture change	Indirectly capture via changes in projected area, height/width, compactness	Change^−^ spatial masks, Dispersion^+^ (when reorientation/occlusion causes spatially structured disappearances/appearances)
Spatially localized turnover (growth vs. occlusion vs. loss)	Not directly quantified by standard scalar traits (area/height/width/compactness/color) because they produce pixel-level change maps between timepoints	Change^+^/Change^−^ (explicit pixel-level additions removals), MaxChange^+^/MaxChange^−^
Single-modality thermal stress state	Mean canopy/leaf temperature (IR imaging) [51]	Not quantifiable
Single-modality photosynthetic stress state	Fluorescence indices (e.g., chlorophyll fluorescence efficiency measures) [52]	Not quantifiable
Cross-modality physiological coupling/decoupling under drought	Not directly quantified; inferred only by manual correlation of separate traits (e.g., area vs. temperature vs. fluorescence)	IC and IMI
Multimodal “coupling” or “decoupling” under drought	Assessed post hoc by comparing separate traits (e.g., area vs. temperature vs. fluorescence) [51]	IC and IMI
Architectural asymmetry across viewpoints	Multi-view images commonly used, but conventional traits typically report single-view metrics or aggregated size proxies; explicit “min/max views” asymmetric descriptors are not standard outputs [9,17,49]	TAR_min_, TAR_max_, and TWR

## Data Availability

The SIMID and SIPID dataset utilized and created in this study is publicly available and accessible at the following link: https://doi.org/10.5281/zenodo.17400167 (accessed on 21 October 2025).
